# Differential Expression Analysis Identifies Candidate Synaptogenic Molecules for Wiring Direction-Selective Circuits in the Retina

**DOI:** 10.1523/JNEUROSCI.1461-23.2024

**Published:** 2024-03-21

**Authors:** Joshua M. Tworig, Ryan D. Morrie, Karina Bistrong, Rachana D. Somaiya, Shaw Hsu, Jocelyn Liang, Karen G. Cornejo, Marla B. Feller

**Affiliations:** ^1^Department of Molecular and Cell Biology, University of California Berkeley, Berkeley, California 94720; ^2^Helen Wills Neuroscience Institute, University of California Berkeley, Berkeley, California 94720

**Keywords:** direction selectivity, retina, retinal ganglion cell, RNA-seq, two-photon calcium imaging

## Abstract

An organizational feature of neural circuits is the specificity of synaptic connections. A striking example is the direction-selective (DS) circuit of the retina. There are multiple subtypes of DS retinal ganglion cells (DSGCs) that prefer motion along one of four preferred directions. This computation is mediated by selective wiring of a single inhibitory interneuron, the starburst amacrine cell (SAC), with each DSGC subtype preferentially receiving input from a subset of SAC processes. We hypothesize that the molecular basis of this wiring is mediated in part by unique expression profiles of DSGC subtypes. To test this, we first performed paired recordings from isolated mouse retinas of both sexes to determine that postnatal day 10 (P10) represents the age at which asymmetric synapses form. Second, we performed RNA sequencing and differential expression analysis on isolated P10 ON–OFF DSGCs tuned for either nasal or ventral motion and identified candidates which may promote direction-specific wiring. We then used a conditional knock-out strategy to test the role of one candidate, the secreted synaptic organizer cerebellin-4 (Cbln4), in the development of DS tuning. Using two-photon calcium imaging, we observed a small deficit in directional tuning among ventral-preferring DSGCs lacking Cbln4, though whole-cell voltage-clamp recordings did not identify a significant change in inhibitory inputs. This suggests that Cbln4 does not function primarily via a cell-autonomous mechanism to instruct wiring of DS circuits. Nevertheless, our transcriptomic analysis identified unique candidate factors for gaining insights into the molecular mechanisms that instruct wiring specificity in the DS circuit.

## Significance Statement

By performing mRNA transcriptome analysis on three populations of direction-selective ganglion cells (DSGCs)—two preferring horizontal motion and one preferring vertical motion—we identified differentially expressed candidate molecules potentially involved in cell subtype-specific synaptogenesis within this circuit. We tested the role of one differentially expressed candidate, cerebellin-4 (Cbln4), enriched in ventral-preferring DSGCs. Using a targeted knock-out approach, the deletion of Cbln4 led to a small reduction in direction-selective tuning while maintaining dendritic morphology and normal strength and asymmetry of inhibitory synaptic transmission. Overall, we have shown that this approach can be used to identify interesting candidate molecules, and future functional studies are required to reveal the mechanisms by which these candidates influence synaptic wiring within specific circuits.

## Introduction

As the brain develops, diverse populations of neurons form precise wiring arrangements which enable sensation, perception, and behavior. Throughout neural development, multiple neuronal classes defined by their gene expression profiles and morphologies intermingle as they find their appropriate pre- and postsynaptic partners, a process referred to as synapse specificity (Sanes and Zipursky, 2020). In the mammalian retina, over 40 types of output neurons—retinal ganglion cells (RGCs)—must synapse with >60 types of amacrine cells and 15 types of bipolar cells to form functional circuits tuned for various visual features such as contrast, orientation, and motion (Shekhar et al., 2016, 2022; Tran et al., 2019; Yan et al., 2020; Goetz et al., 2022). During retinal development, there are two steps to this process. First, the neurites of pre- and postsynaptic neurons arborize into distinct sublayers within a nascent inner plexiform layer (IPL) using a molecular code composed of multigene families of transmembrane or secreted proteins (Yamagata and Sanes, 2008; Sun et al., 2013; Visser et al., 2015; Peng et al., 2017; Duan et al., 2018). Second, pre- and postsynaptic neurons within IPL sublayers find their appropriate partners and reject inappropriate ones (reviewed in Hoon et al., 2014; Hamilton et al., 2021). Although the molecules governing laminar organization of the retina have been well studied, the molecular determinants of synapse specificity within the plane of a single lamina remain an open question.

The retinal direction-selective circuit is an ideal model circuit for studying synapse specificity. It is composed of the radially symmetric starburst amacrine cell (SAC) and its postsynaptic partner, the direction-selective ganglion cell (DSGC). SACs release the inhibitory neurotransmitter GABA onto DSGCs in a direction-selective manner, with greater GABA release during motion from each proximal to distal SAC process than motion in the opposite direction (Euler et al., 2002; Vlasits et al., 2016). This dendritic direction selectivity, paired with a wiring arrangement in which DSGCs preferentially form synapses with SAC processes oriented in the DSGC’s null direction, confers direction selectivity onto the firing of ON and ON–OFF DSGCs (Briggman et al., 2011; Ding et al., 2016). DSGCs cluster into four cardinal preferred directions—nasal, temporal, ventral, and dorsal—and these directional clusters express distinct molecular markers (Kerschensteiner and Hardesty, 2022). The precise and stereotyped wiring arrangement between SAC processes and DSGCs emerges during the second postnatal week of development, prior to eye-opening in mice (Wei et al., 2010; Yonehara et al., 2010; Morrie and Feller, 2015) and persists in the absence of visually evoked activity (Tiriac et al., 2022). This suggests that an instructive molecular signal exists to promote synaptogenesis between DSGCs and the appropriate SAC processes during this short period of development.

The molecular determinants that underlie wiring differences between the different DSGC subtypes and SACs are unknown. To identify such determinants, we performed an RNA sequencing-based screen for differentially expressed genes among three genetically identified ON–OFF DSGC subtypes: two nasal motion preferring and one ventral motion preferring. We report the transcripts enriched in ventral or nasal motion-preferring DSGCs during the emergence of asymmetric SAC→DSGC wiring, and we follow up on one candidate synaptogenic molecule, cerebellin-4 (Cbln4), to assess its role in synapse specificity in the direction-selective circuit.

## Materials and Methods

### Animals

All mice were maintained on mixed C57BL/6 backgrounds. Mice of both sexes aged postnatal days (P) 15–35 were used in this study. All animal procedures were approved by the UC Berkeley Institutional Animal Care and Use Committee and conformed to the NIH Guide for the Care and Use of Laboratory Animals, the Public Health Service Policy, and the SfN Policy on the Use of Animals in Neuroscience Research.

*Drd4-GFP* and *Trhr-GFP* mice were obtained from Mutant Mouse Regional Resource Centers (MMRRC; http://www.mmrrc.org/strains/30036/030036.html and http://www.mmrrc.org/strains/231/0231.html, respectively; Gong et al., 2003) and express GFP in nasal-preferring ON–OFF DSGCs (Rivlin-Etzion et al., 2011). *Hb9-GFP* (JAX strain 005029) mice express GFP in ventral-preferring ON–OFF DSGCs (Trenholm et al., 2011). Because the *Hb9-GFP* and *Trhr-GFP* lines express GFP in some cone photoreceptors and amacrine cells, respectively, we had to colabel DSGCs with tdTomato to enable high confidence separation of DSGCs from other GFP-expressing cells during sorting. We achieved this by crossing *Trhr-GFP* and *Hb9-GFP* mice with *VGlut2-Cre* (JAX strain 028863) and Ai9/tdTomato mice (JAX strain 007909). In these crosses, *VGlut2-Cre* is expressed in nearly all RGCs and a few cells in the outer nuclear layer (Morrie, 2018).

To target SAC→DSGC pairs, ChAT-Cre;nGFP;Trhr mice were generated by crossing together three mouse lines: (1) *ChaT-Cre* (JAX strain 006410), with Cre driven by the endogenous choline acetyltransferase promoter, (2) nuclear GFP (JAX strain 008606), with a nuclear-localized GFP-lacZ function protein downstream of a loxP-flanked STOP sequence, and (3) *Trhr-GFP* mice.

To visualize the formation of SAC varicosities, we induced sparse tdTomato expression in SACs by delivering tamoxifen to ChAT-Cre:tdTomato mice via intraperitoneal injection at a dose of 200 µg in 125 µl sunflower oil at P4.

To knock out *Cbln4* from RGCs, we obtained a strain carrying a conditional reporter/knock-out allele, *Cbln4^fl^*, (JAX strain 032960) and crossed it with the *VGlut2-Cre* (JAX strain 028863) line, which enabled RGC-specific *Cbln4* knock-out mediated by Cre recombinase (Ellis et al., 2016; Seigneur and Südhof, 2017). In the absence of Cre, *Cbln4^fl/fl^* cells express functional Cbln4 and mVenus from the same mRNA via an internal ribosome-entry site (IRES), enabling labeling and identification of Cbln4+ cells in live tissue. In Cre-expressing cells, exons 2 and 3 of *Cbln4* are excised, and sequence encoding tdTomato is placed in-frame with the truncated *Cbln4* allele such that *Cbln4^−/−^* cells express tdTomato. Expression of mVenus or tdTomato was used to target Cbln4-positive or Cbln4-knock-out RGCs for whole-cell recording and morphological assessment. In a subset of experiments, we crossed these mice with *Hb9-GFP* mice to robustly label a subpopulation of ventral DSGCs.

### Retinal preparation

Animals of either sex were anesthetized via isoflurane inhalation and decapitated. Eyes were enucleated and retinas dissected in oxygenated (95% O_2_/5% CO_2_) Ames medium at room temperature under bright-field (for retinal dissociations) or infrared (for experimental recordings) illumination. For recording and imaging experiments on whole-mount samples, isolated retinas were mounted ganglion cell side up on filter paper (Millipore) and transferred into the recording chamber of an upright microscope for imaging and electrophysiological recording. Retinas were continuously superfused with oxygenated Ames (2–4 ml/min) at 32–34°C for the duration of experiments and kept in the dark at room temperature in oxygenated Ames when not imaging or recording.

### Fluorescence-activated sorting of DSGCs

P10 retinas were dissociated as in Kay et al. (2011) with some modifications. Before beginning dissections, 80 μl of papain solution (1 U/µl; Worthington LS003126) was activated in 5 ml of HBSS plus HEPES (10 mM) by adding DNase I (50 µl, 13.33 U/µl; Sigma D4527) and ʟ-cysteine (50 µl, 152 mM; Sigma C1276). This solution was sterile filtered and left at 37°C during retinal dissection. Retinas were dissected in cold HBSS plus HEPES, and whole retinal cups were stored in fresh HBSS plus HEPES on a cold block until all retinas were dissected. Retinas were then incubated in activated papain solution for 21 min at 37°C with gentle shaking every 7 min. Retinas were spun for 1.5 min at 200 g, and then papain solution was aspirated and replaced with 2 ml of ovomucoid solution (Worthington LS003087). Retinas were broken up via trituration with a P1000 tip and spun for 20 s at 200*×g*, and supernatant containing single cells was transferred to a new tube. An additional 1 ml of lo ovomucoid was added to the dissociation mixture, and the trituration process was repeated until all tissue was dissociated into a single cell suspension. This product was passed through a 70 μm filter and spun down at 375*×g* for 10 min. The supernatant was aspirated, and cell pellets were resuspended in 2 ml of MEM-B [4% bovine serum albumin (BSA; Sigma A9418) in MEM without glutamine (Invitrogen 11090)]. Three biological replicates were obtained for each DSGC RNA-seq dataset.

GFP-positive or GFP/tdTom-double-positive cells were sorted via fluorescence-activated cell sorting (FACS) using a BD FACSAria Fusion cytometer. To draw gates, a GFP/tdTom-negative sample was run through the machine, and >1,000,000 events were recorded. Cells were selected based on size (largest cells for RGCs), doublets were excluded, and GFP+ or GFP/tdTom+ cells were collected based on these gates. Cells were sorted into 500 µl of TRIzol LS (Invitrogen 10296010), and cDNA libraries were prepared using Smart-Seq technology by the Berkeley Functional Genomics Laboratory.

### RNA sequencing and differential expression analysis

Following RNA isolation, quality assessment, and cDNA library preparation, we carried out RNA sequencing using an Illumina HiSeq 4000 system, which generated 100-base-pair paired-end reads. We performed quality assessment and preprocessing on sequence reads using FastQC and Trimmomatic (Andrews, 2010; Bolger et al., 2014). Reads were aligned and mapped to the mouse genome using Hisat2 (Kim et al., 2019) or pseudoaligned to the mouse transcriptome using Kallisto (Bray et al., 2016). We used the Genome Reference Consortium Mouse Build 38/mm10 assembly for these alignments. This dual alignment approach enabled identification of candidate molecules at the gene as well as splice isoform level. After generating genomic alignments with Hisat2, we used Subread's featureCounts function to count fragments from paired-end reads (Liao et al., 2014). On average, featureCounts processed 64.56 ± 11.90 million fragments per sample, and 77.8 ± 0.5% of these fragments were successfully aligned to the genome. When processed and counted using Kallisto, an average of 64.7± 1.1% of 50.46 ± 11.58 million reads were successfully aligned to the transcriptome.

After obtaining high-quality sequence alignments and counts, the counts were normalized and tested for differential expression between DSGCs of orthogonal directional preference using DESeq2 (Love et al., 2014) for reads aligned to the genome and counted using featureCounts or Sleuth (Pimentel et al., 2017) for reads aligned to the transcriptome and counted using Kallisto. The described results originate from our Kallisto/Sleuth quantification of reads aligned to the transcriptome, as this method identified many of the same genes as those identified using a gene-level analysis, in addition to some differentially expressed alternatively spliced transcripts. Kallisto produces estimates of transcript level counts as it is possible for reads to be mapped to multiple gene isoforms. As such, we report and perform statistical testing on count estimates. For visualization of gene expression in heatmaps, estimated counts were log_2_ normalized. Dot plots from single-cell (scRNA-seq) expression data were visualized and exported using Single Cell Portal [Broad Institute; https://singlecell.broadinstitute.org/single_cell; GEO accession GSE185671 (Shekhar et al., 2022), GSE137400 (Tran et al., 2019), GSE149715 (Yan et al., 2020)].

We directed Sleuth to perform the Wald test to check for differential expression according to directional preference and to output β values to represent effect sizes for expression differences between horizontal and vertical motion-preferring DSGCs. *p* values for individual genes were corrected for multiple testing using the Benjamini–Hochberg method. We used an adjusted *p* value cutoff of 0.01 and a β value of >2 or less than −2 to generate lists of up- and downregulated transcripts. Gene Ontology (GO) enrichment analysis was performed using GO::TermFinder, an open source platform for genome-wide analysis of gene function (Boyle et al., 2004). This tool identifies significantly enriched GO terms relative to a gene background, which was the set of genes with total estimated counts >5 when summed across all samples.

As a supplementary analysis, we also performed pairwise differential expression tests on transcripts from Drd4-GFP versus *Hb9-GFP*, *Trhr-GFP* versus *Hb9-GFP*, and *Trhr-GFP* versus Drd4-GFP. After applying the same adjusted *p* value and β value cutoffs, we intersected the sets of differentially expressed transcripts generated from tests of Drd4-GFP and *Trhr-GFP* versus *Hb9-GFP* to identify candidates related to nasal versus ventral DSGC wiring specificity.

### In situ hybridization and immunohistochemistry

Single molecule RNA in situ hybridization for Figure 2*B* was carried out using the RNAscope Multiplex Fluorescent protocol (Advanced Cell Diagnostics). Eyes dissected from P10 *Trhr-GFP*, Drd4-GFP, and *Hb9-GFP* mice were fixed in 4% paraformaldehyde (PFA) overnight at 4°C, followed by cryopreservation in 30% sucrose. Retinal slices were cut using a microtome set to a thickness of 30 μm. Slices were postfixed in 4% PFA, dehydrated in ethanol series, dried, and pretreated with RNAscope target retrieval reagents and protease before hybridizing the probe for Cbln4 mRNA (Mm-Cbln4-C3). After carrying out signal amplification steps, HRP-based fluorescent detection was performed using a fluorescently tagged Opal dye (PerkinElmer). Then, we stained for GFP in the same samples by washing them in TBST, followed by blocking for 30 min at room temperature in TBS with 10% normal donkey serum and 1% BSA. Samples were then incubated in chicken anti-GFP primary antibody (ab13970; 1:1,000 in TBS-1% BSA) for 2 h at room temperature. After a series of TBST washes, secondary antibody (1:1,000 in TBS-1% BSA) with conjugated Alexa fluorophore was applied for 45 min at room temperature.

For co-staining Cbln4 mRNA-expressing cells with immunohistochemistry against RBPMS protein (Fig. 4*A–C*), eyes dissected from P10 to P11 control and mutant mice were fixed in 4% PFA overnight at 4°C and 30% sucrose for 3 d. Retinal slices were cut using a cryostat set to a thickness of 16 μm. Standard RNAscope Multiplex Fluorescent protocol was used to detect Cbln4 mRNA, and the signal was amplified using a fluorescently tagged Opal dye (PerkinElmer) as described above. These slides were then blocked for 30 min at room temperature in blocking solution containing 1% BSA and 0.1% Triton X in TBS. Retinal slices were then incubated with a rabbit antibody against RBPMS (1:500, Abcam ab152101) overnight at 4°C. The next day, slides were washed in 0.1% Tween20 and 0.1% Triton X in TBS solution and incubated at room temperature for 2 h with Alexa Fluor 647 goat anti-rabbit (1:500, Jackson ImmunoResearch Laboratories, 111-605-003). After a series of washes with 0.1% Tween20 and 0.1% Triton X in TBS solution to remove background signal, DAPI was applied to slices prior to mounting with ProLong Gold antifade mounting medium.

Slices were imaged on a confocal scanning microscope (Zeiss LSM 880 NLO AxioExaminer equipped with Diode 405 nm, DPSS 561, HeNe594, and HeNe933 lasers, Molecular Imaging Center at UC Berkeley) with a 63×/1.4NA Plan-Apochromat oil immersion objective (pixel size, 0.07 μm^2^). *Z* stacks with 1 μm step size were used to acquire images of DAPI (excitation, 405 nm; emission window, 410–505 nm), Opal 570-labeled Cbln4 mRNA (excitation, 647 nm; emission peak, 650 nm), and RGCs labeled with Alexa-568 secondary (excitation, 561 nm; emission peak, 603 nm). Stacks were bandpass filtered to reduce high-frequency noise and normalize background fluorescence. mRNA puncta which were colocalized with RBPMS antibody were manually counted at each *Z* section, for each RBPMS+ soma. Stacks were then reconstructed using maximum intensity projections in FIJI/ImageJ for presentation (Schindelin et al., 2012).

### Population two-photon calcium imaging

For population two-photon imaging of RGCs, *Hb9-GFP;Cbln4^fl/fl^* or *Hb9-GFP;*vGlut2-Cre;*Cbln4^fl/fl^* retinas were first bolus loaded with Cal-590. Two-photon fluorescence measurements were obtained with a modified movable objective microscope (MOM; Sutter Instrument) equipped with a Nikon 16X, 0.80 NA, N16×LWD-PF objective (Nikon). Fluorescence excitation was evoked with an ultrafast pulsed laser (Chameleon Ultra II; Coherent) tuned to 920 nm for GFP or 1,040 nm for Cal-590. The microscope system was controlled by ScanImage software (www.scanimage.org). Scan parameters for imaging during visual stimuli were as follows (pixels/line × lines/frame [frame rate in Hz]): 256 × 256 (2.96 Hz), at 1 ms/line. The microscope was equipped with through-the-objective light stimulation. Methods previously described in (Tiriac et al., 2022).

#### Visual stimulation for calcium imaging

For simultaneous visual stimulation and calcium imaging, to decrease the background signal entering the photomultiplier tubes due to UV light stimulation, the stimuli were delivered on the flyback of the fast axis scanning mirror during a unidirectional scan to interleave the stimuli with the imaging. The rate at which the visual stimulus was shown with the interline (1 KHz) is faster than the flicker fusion frequency for mice (∼15–20 Hz; Tanimoto et al., 2009). During simultaneous imaging, the bar stimulus was a rectangle (width, 500 μm; length, 1,000 μm) that drifted across the field of view in eight different directions (every 45°) at speed of 250 µm/s. The intensity of the UV stimulus was 2 × 10^6^ photons s^−1^ µm^−2^ on a dark background (dark background, 2 × 10^3^ photons s^−1^ µm^−2^).

#### Processing of calcium imaging data

Raw movies were motion corrected and normalized into Δ*F* / *F*_0_ automatically using a custom-made FIJI macro that was run in ImageJ v1.53q. Briefly, (1) movies were motion corrected using the “correct 3D drift” plugin in FIJI on a duplicate of the raw data that had been averaged in the time dimension (zMean, 30 s)—note that this step can only correct *x*–*y* drift and *z* drift cannot be corrected and hence was prevented during acquisition. (2) Frames where the light stimuli occurred were removed to isolate baseline *F*. (3) The baseline *F* was subtracted from the raw *F* movie, and this result was divided by the baseline *F*. The resulting Δ*F* / *F*_0_ movies were then transferred to MATLAB for further image analysis.

#### Semi-automatic detection of DSGCs from calcium imaging

A MATLAB code was written to automatically identify potential ROIs within a Δ*F* / *F*_0_ movie that were direction selective. Briefly, the code analyzes every pixel in the *x*–*y* dimension by (1) taking a mean average of its neighbor pixels, (2) computing the average peak Δ*F* / *F*_0_ response for every stimulus direction, and (3) computing the vector sum and direction selectivity index (DSI) for each pixel:

$$\mathrm{vector}\;\mathrm{sum}=\;\frac{\sum_{n=1}^{8\;\mathrm{directions}}\;(x_{n},y_{n}\;)}{\sum_{n=1}^{8\;\mathrm{directions}}\;\mathrm{mean}\left(\mathrm{peak}\left(\frac{\Delta F}{F_{0,n}}\right)\right)},$$

where *x_n_* and *y_n_* are the Cartesian coordinates of the polar vector where the direction of the vector is the stimulus direction, and the length of the vector is the average peak Δ*F* / *F*_0_ for that direction. The direction of the vector sum is the ROI's preferred direction, which is used to calculate DSI, and the length of the vector sum is the magnitude of the tuning:

$$\mathrm{Direction}\;\;\mathrm{selectivity}\;\mathrm{index}=\frac{\left(\frac{\Delta F}{F_{0,\mathrm{pref}}}-\frac{\Delta F}{F_{0,\mathrm{null}}}\right)}{\left(\frac{\Delta F}{F_{0,\mathrm{pref}}}+\frac{\Delta F}{F_{0,\mathrm{null}}}\right)},$$

where pref is the direction angle closest to the vector sum's direction and null is 180°C rotated from pref.

Next the MATLAB code used a 2D median filter to enrich the cell-like ROIs that exhibit similar preferred directions. The resulting image is then overlaid on an average fluorescence image of the motion-corrected movie and oval ROIs are drawn in FIJI over regions that were mathematically determined to be direction selective and that also correspond to an anatomical cell. These ROIs are then transferred to MATLAB for further analysis.

Three criteria were used to categorize DSGCs. The numbers of recorded cells that passed these criteria are shown in Table 1.

**Table 1.** Summary table for populations included in Figure 5.

	WT #	KO #
Total number Hb9-GFP+ cells	412	373
Hb9-GFP+ cells that pass criteria 1 (Fig. 5*C*)	246	238
Hb9-GFP+ cells that pass criteria 1 and 2 (Fig. 5*D*)	73	56
Total number of cells that pass criteria 1 and 3 (Fig. 5*E*)	365	253
Hb9-GFP+ cells that pass criteria 1, 2, and 3 (Fig. 5*F*)	48	32

Three criteria were used: (1) robust calcium responses that are consistent across three trials; (2) classified as significantly direction selective, as determined by a permutation test with a 95% threshold; and (3) fall into the ventral-preferring cluster as determined by a *k*-means test.

#### Manual classification of ON–OFF cells from calcium imaging

A MATLAB code was written to present a user with a GUI that cycled between every identified cell. The GUI was customized to display the chronological Δ*F* / *F*_0_ trace of the cell, the three blocked and averaged Δ*F* / *F*_0_ traces for each of the eight different directions, and a tuning plot of the cell using the blocked traces. Using this GUI, a user classified cells as either “On–Off” if they exhibited a Δ*F* / *F*_0_ peak at the onset and offset of the moving bars or “On” if they exhibited a Δ*F* / *F*_0_ peak only at the onset of the moving bars. The first criteria for DSGC classification was whether the cells exhibited consistent responses to each of the three trials for the eight different directions.

#### Statistical determination of direction selectivity from calcium imaging

The second criteria was based on a statistical approach to determine which cells were significantly direction selective: For each cell, the DSI was first calculated. Then, for 1,000 permutations in silico, the directions of the moving bar stimuli were block shuffled and the DSI was again calculated. The cell's DSI calculated from the nonpermuted dataset was ranked against all the DSIs calculated from the permuted dataset. If the cell's actual DSI ranked higher than 95% of the permuted DSIs, it was determined to be significantly direction selective.

#### Clustering analysis from calcium imaging

The third criteria involved classifying the preferred direction of DSGCs. We used the same clustering method that was described in a previous study (Bos et al., 2016). Briefly, *k*-means clustering analysis in MATLAB software was used to evaluate the pattern of distribution of the preferred directions from On–Off DSGCs for which we did not have a genetic label. All the lengths of the preferred directions were fixed to 1, and these were transformed into Cartesian coordinates for subsequent angular distance measurement. This method optimizes the set of clusters with respect to the distance between each point and the centroid of its cluster, summed for all points. We compared 2–8 cluster numbers, and we calculated the fitness of clustering by using the silhouette value (SV):

$$\mathrm{SV}=\;\frac{\left(b(i)-a(i)\right)}{(a(i),b(i))\;}\;$$

where *a*(*i*) is the average distance between *i* and all other data within the same cluster (called measure of cohesion), and *b*(*i*) is the average distance between *i* and all points in the nearest cluster (called measure of separation from the closest other cluster). An SV close to 1 indicates data perfectly clustered, whereas an SV close to 0 reflects data which are ambiguously clustered.

Since a subset of experiments were performed in transgenic mice where known DSGCs were labeled with GFP, we used these known cell types to define the clusters. For example, the cluster that pointed ventrally and matched the *Hb9-GFP*, which labels a subset of ventral-preferring cells, was defined to be the ventral cluster of DSGCs. The cluster 180°C rotated from the defined ventral cluster was defined as the dorsal cluster, and the cluster 180°C rotated from the nasal cluster was defined as the temporal cluster.

### Targeted whole-cell electrophysiology

Cbln4 reporter/knock-out RGCs in *Cbln4^fl/fl^* or *VGlut2-Cre;Cbln4^fl/fl^* retinas were targeted under two-photon illumination using a modified MOM (Sutter Instrument) equipped with a 60×, 1.0 NA, LUMPlanFLN water immersion objective (Olympus America). Two-photon excitation was evoked with an ultrafast pulsed laser (Chameleon Ultra II; Coherent) tuned to 950 nm (for mVenus-expressing Cbln4+ RGCs) or 1,040 nm (for tdTomato-expressing Cbln4-KO RGCs). The microscope was controlled by ScanImage software (www.scanimage.org). Scan parameters during soma targeting were 512 × 512 pixels at 1 ms/line, with 1.48 Hz frame rate. Low expression levels of mVenus and tdTomato in live samples required us to average 5 or more frames to clearly identify reporter-positive somas for targeting. After recording visual responses from neurons and allowing the cell to dialyze with fluorescent dye (Alexa-594), we switched to a 16×, 0.8NA, LWD water immersion objective (Nikon) and to 810 nm excitation to acquire *Z* stacks through dye-filled somas and dendrites for later reconstruction and morphological assessment. *Z* stacks through the ganglion cell layer (GCL) and IPL were taken at 4 frames per slice, 512 × 512 pixels, 1 ms/line.

Whole-cell voltage-clamp recordings were made from reporter-expressing RGCs in *Cbln4^fl/fl^* or *VGlut2-Cre;Cbln4^fl/fl^* retinas. After identifying the target cell under two-photon illumination, we switched to widefield infrared illumination to patch onto its soma with a glass microelectrode with resistance of 3–5 MΩ (PC-10 pipette puller; Narishige) filled with an internal solution containing the following (in mM): 110 CsMeSO_4_, 2.8 NaCl, 20 HEPES, 4 EGTA, 5 TEA-Cl, 4 Mg-ATP, 0.3 Na_3_GTP, 10 Na_2_ phosphocreatine, and 5 QX-Cl, pH 7.2 (290 mOsm). Electrodes also contained ∼25 μM Alexa-594 hydrazide to facilitate morphological imaging. The liquid junction potential correction for this solution was −10 mV. Signals were acquired using pClamp10 recording software and a MultiClamp 700A amplifier (Molecular Devices), sampled at 20 kHz and low-pass filtered at 2 kHz. During each recording, input resistance was monitored, and a current–voltage relationship was measured to ensure quality of the recording. Synaptic responses to visual stimuli were recorded while applying a holding voltage of either +12 or −60 mV to measure inhibition or excitation, respectively.

#### Paired recordings

Paired recordings of SACs and DSGCs were performed as previously described (Morrie and Feller, 2015). Oriented retinas were placed under the microscope in oxygenated ACSF containing the following excitatory neurotransmitter blockers (in mM): 0.05 AP-5, 0.02 DNQX, and 0.008 DHβE. GFP+ DSGCs and nGFP+ SACs located on each DSGC's null or preferred side were identified under two-photon illumination and targeted as above. Whole-cell voltage-clamp recordings were achieved for a given DSGC before targeting SACs. Recordings from DSGCs were performed as above, but for SACs the internal solution EGTA concentration was 0.1 mM. To calculate SAC→DSGC synaptic conductances, DSGCs were held at voltage potentials ranging from −100 to +20 mV, while SACs were depolarized three times from the holding potential (−70 mV) to 0 mV for 50 ms.

Inhibitory conductance analysis of paired SAC→DSGC recordings was performed in IGOR Pro (described in Taylor and Vaney, 2002). Briefly, sweeps at each DSGC holding potential were averaged and then the baseline holding current (defined as the average current prior to SAC stimulation) was subtracted from each average trace. We compensated for the series resistance (*R*_s_) by measuring the series and input resistance (*R*_in_) from a −5 mV pulse at the end of each trace. We used the following equations for compensation of the recorded current (*I_m_*) and the holding potential (*V_h_*):

$$I_{\mathrm{syn}}(t)\;=\;\frac{\left(R_{\mathrm{in}}+R_{s}\right)}{R_{\mathrm{in}}}\;*\;I_{m}(t),$$

$$V(t)\;=V_{h}\;-\;I_{m}(t)\;*\;R_{s}.$$

Then we fit a line to the IV data (*I*_syn_ vs *V*) for the various holding potentials at each time point (*t*) in the trace. The slopes and intercepts of these lines were used to calculate the inhibitory conductance gT (the slope) and the reversal potential *V*_rev_ (−intercept/slope). We controlled for quality of recording by requiring an *R*^2^ value for the linear fit of the IV data above 0.85.

#### Visual stimulation for electrophysiology

Visual stimuli consisted of static spots or drifting bars centered over the dendritic arbor of each recorded cell and focused on photoreceptors using the Olympus 60×, 1.0 NA, LUMPlanFLN objective. Stimulus patterns were generated in MATLAB using the Psychophysics Toolbox and displayed onto the retina using a UV (375 nm) LED projected through a digital micromirror device. The intensity of the UV stimulus was 2 × 10^6^ photons s^−1^ µm^−2^ on a dark background (dark background, 2 × 10^3^ photons s^−1^ µm^−2^). All stimuli were displayed in triplicate. For static stimuli, spots of variable diameter (0–440 μm) were pseudorandomly flashed for 1–2 s while recording postsynaptic currents in gap-free mode, with a 1 s intertrial delay. For drifting bar stimuli, 150-μm-wide rectangles drifted across the receptive field of each recorded RGC in eight different directions and at two different speeds (250 μm/s and 1,000 μm/s). The length of the bar was adjusted such that onset and offset postsynaptic currents could be analyzed separately (250 μm for slower bars, 1,000 μm for faster bars). Each direction was repeated three times in block shuffled manner, such that each direction played once before repeating, with 2 s between individual stimulus sweeps.

#### Quantification of visual response properties from electrophysiology recordings

For all stimuli sizes, directions, and speeds, we measured the peak onset and offset E/IPSC, and we used these measures to calculate the tuning properties of each recorded Cbln4+ or Cbln4-KO cell. For static spots, we calculated the ON and OFF center-surround (CS) index as follows:

$$\mathrm{CS}\;\;\mathrm{index}=1-\frac{R_{\mathrm{large}\;\mathrm{spot}}}{R_{\mathrm{all}\;\mathrm{spots}}},$$

where $R_{\mathrm{large}\;\mathrm{spot}}$ refers to the peak current response to the largest spot (440 μm) and $R_{\mathrm{all}\;\mathrm{spots}}$ refers to the maximum response magnitude across all spot diameters (up to and including 440 μm). The CS index is close to zero if the response to the large spot is close to the maximum response across all sizes (indicating little to no surround suppression) and approaches one as the response to the large spot diminishes due to surround suppression.

We calculated the DSI for postsynaptic currents in response to moving bars by first calculating a vector sum from the response amplitudes in all directions:

$$R_{x}=\overset{n}{\underset{i=1}{\sum }}\;R_{i\;}\cdot \cos\;\cos\;\theta_{i},$$

$$R_{y}=\overset{n}{\underset{i=1}{\sum }}\;R_{i\;}\cdot \sin\;\sin\;\theta_{i},$$

$$R_{t}=\overset{n}{\underset{i=1}{\sum }}\;R_{i},$$

$$R^{⇀}=\sqrt{R_{x}^{2}+R_{y}^{2}},$$

$$\theta^{⇀}=\frac{R_{y}}{R_{x}},$$

where $R_{i}$ refers to the peak response for the $i^{\mathrm{th}}$ stimulus direction (θ), $R_{x}$ and $R_{y}$ are the *x* and *y* components of the response, and $R_{t}$ is the summation of all responses and was used to calculate a normalized vector sum. The direction of this vector sum was used to determine the “preferred” stimulus direction for these currents. When recording from ON–OFF DSGCs, the “preferred” direction for IPSCs corresponds to the null direction of the cell. After measuring the amplitude of the preferred direction current, the “null” direction current is the average response measured during stimuli moving in the 180° opposite direction. DSI was then calculated as follows:

$$\mathrm{DSI}=\;\frac{R_{\mathrm{PD}}-R_{\mathrm{ND}}}{R_{\mathrm{PD}}+R_{\mathrm{ND}}},$$

where $R_{\mathrm{PD}}$ refers to the peak response in the preferred direction and $R_{\mathrm{ND}}$ refers to the peak response in the null direction. DSI typically ranges from 0 to 1 with values closer to one indicating strong directional tuning and values close to zero indicating symmetric responses.

We calculated the speed tuning index (SI) of all recorded cells in an analogous manner:

$$\mathrm{SI}=\;\frac{R_{\mathrm{fast}}-R_{\mathrm{slow}}}{R_{\mathrm{fast}}+R_{\mathrm{slow}}},$$

where $R_{\mathrm{fast}}$ refers to the peak response to fast bars and $R_{\mathrm{slow}}$ refers to the peak response to slow bars. SI typically ranges from +1 to −1 with positive values indicating preference for fast motion and negative values indicating preference for slower motion.

### SAC varicosity analysis

Whole-mount retinas were transferred onto filter paper and fixed in 4% PFA solution for 20 min at room temperature and then washed in block solution (2% donkey serum, 2% BSA, 0.3% Triton X, 0.2% sodium azide in PBS, 5 × 20 min) at room temperature. Next, retinas were incubated in primary antibody (1:500 rabbit anti-DsRed) for 72 h at 4°C and then washed in block solution for 72 h at 4°C. The retinas were then incubated in secondary antibody (1:1,000 goat anti-rabbit Alexa-488, 1:1,000 goat anti-rabbit Alexa-594) for 72 h at 4°C and washed in block solution for 72 h at 4°C. Then the retinas were mounted onto slides and coverslipped with fluoromount-G (SouthernBiotech).

SACs were imaged using a Zeiss LSM AxioObserver laser scanning confocal microscope with Zen 2010 software located at the University of California, Berkeley Molecular Imaging Center. Fluorescent confocal image stacks were acquired with a Plan-Apochromat 40× (NA = 1.4) or a Plan-Apochromat 60× (NA = 1.4) oil immersion objective and Immersol 518F oil. Alexa-488 was excited using Argon 488 laser at ∼15% transmission, and emission was detected from 493 to 630 nm. Alexa-594 was excited using HeNe 594 laser at ∼20% transmission, and emission was detected from 599 to 734 nm. Images were taken at frame size of 2,048 × 2,048 pixel with a pixel size of ∼100 nm and a pixel dwell time of ∼3.15 µs. Pinhole size was set to 1 Airy unit. SAC image stacks were imported into Imaris software (Bitplane), and the dendritic processes and somata were reconstructed in three dimensions using the filament function under the Surpass mode. Varicosities were marked manually using the draw spine feature, and the location and diameter of varicosities were exported using built-in Imaris statistical tools.

### RGC morphology analysis

Image processing for RGC morphology was performed in FIJI/ImageJ (National Institutes of Health; Schindelin et al., 2012). *Z* stacks of filled RGCs were initially bandpass filtered in *x*–*y* space, and multiple slices at each *z* plane were averaged to limit noise and enable quality dendritic reconstructions. Dendrites were traced using the Simple Neurite Tracer plugin in FIJI (Arshadi et al., 2021) to generate reconstructions. Measurements including total number of tips, total process length, and total branch points were derived from traced paths. Paths were converted to binary skeletons to enable segmentation and separate analysis for ON and OFF dendrites. Sholl analysis was performed on dendritic skeletons using concentric rings spaced 5 µm apart (Ferreira et al., 2014). The center of each Sholl radius was placed at the center of the soma, and intersections were counted at each radius overlayed on a maximum projection image of the skeleton. We determined the convex hull area by measuring the area of the smallest convex polygon enclosing the total, ON, or OFF skeleton in an *x*–*y* maximum projection image. Dendritic asymmetry was calculated by first determining the distance between center-of-mass positions for dendrites and soma, and the direction of asymmetry was determined relative to the ventral direction (ventrally oriented dendrites have an orientation of 0°). We calculated a normalized asymmetry index (AI) for each reconstructed cell by extending a line through the soma and dendritic center of mass, with endpoints on the neuron's convex hull border. After calculating the Euclidean distance between each endpoint and the soma, we applied the following equation:

$$\mathrm{AI}=\;\frac{\left|\underline{P_{1}S}-\underline{SP_{2}}\right|}{\underline{P_{1}S}+\underline{SP_{2}}},$$

where $\underline{P_{1}S}$ represents the length of the line segment from one point on the hull border to the soma and $\underline{SP_{2}}$ represents the length of the line segment from the soma to the opposite side of the hull border. Neurons with somas offset from the center of their convex hull exhibit larger AI, independent of the size of their arbor.

To generate projections of skeletons colored by IPL depth, skeleton positions were loaded into MATLAB and plotted using the “pcshow” function.

### Experimental design and statistical analysis

Group measurements are expressed as mean ± SEM unless otherwise indicated. Two-sample *t* tests were used to compare conductances from SAC→DSGC paired recordings. SAC varicosity counts between ages were compared using a Kruskal–Wallis rank sum test followed by pairwise Wilcoxon rank sum tests between ages, with false-discovery rate correction. Differential expression testing was performed using the Wald test. One-way ANOVA was used to test for differences in mRNA puncta across genotypes in situ hybridization images. Sholl intersection profiles between wild-type and Cbln4-knock-out RGCs were compared using two-way repeated measures mixed ANOVA to test for genotype-associated differences in complexity across radii. Two-sample *t* tests were used for comparison of other morphological measurements. Two-way ANOVA was used to test for genotype-associated differences in light response properties and to determine if there is an interaction between Cbln4 genotype and ON versus OFF responses. Post hoc two-sample *t* tests were performed with multiple testing correction using the Benjamini–Hochberg method, where appropriate.

## Results

### Null-oriented SAC processes rapidly form synapses with DSGCs beginning at P10

Paired recordings revealed that SACs undergo a dramatic increase in asymmetric synaptic connections with ON–OFF DSGCs between P7 and P14 (Wei et al., 2010), while optogenetic experiments showed that the emergence of asymmetric connections from SACs to ON DSGCs occurs ∼P8–P9 (Yonehara et al., 2010). To ensure that RNA-seq samples would identify differentially expressed synaptic markers relevant to asymmetric SAC→DSGC wiring, we sought to identify a time point in development in which asymmetric connectivity is emerging using two different metrics.

**Figure 1.**
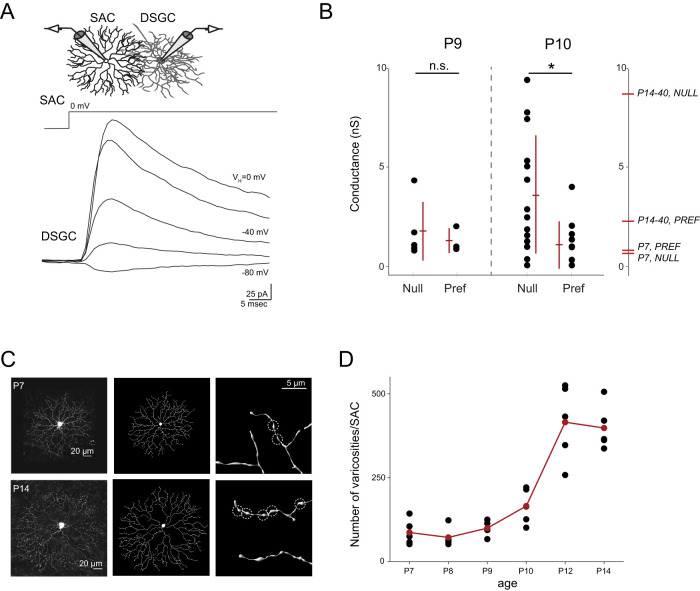
Asymmetric inhibitory SAC→DSGC synaptogenesis emerges beginning at P10. ***A***, Top, Schematic showing paired recording between a SAC and DSGC at P10 in the Chat-Cre;nGFP;*Trhr-GFP* mouse. Bottom, SACs were depolarized to 0 mV while holding DSGCs at the varying potentials (*V*_H_) indicated. Corresponding currents recorded in the DSGC are shown below the voltage step. Depolarization of the SAC induces release of GABA onto the DSGC throughout the duration of the depolarization and during the subsequent tail current in the SAC upon hyper-repolarization. DSGC currents have been leak subtracted. ***B***, Peak inhibitory conductances at P9 and P10 for null and preferred side SAC→DSGC pairs. Inhibitory conductances are small at P9 for all SAC→DSGC pairs. By P10, null-side SAC→DSGC pairs exhibit an increased inhibitory conductance while preferred side pairs do not. *n* = 4 DSGCs at P9, *n* = 5 DSGCs at P10. Multiple preferred- and null-side SACs were recorded from each DSGC. Peak inhibitory conductances from P7 and P14–40 null and preferred side pairs (Wei et al., 2010) are shown on the right *y*-axis. Error bars represent standard deviation. n.s., nonsignificant; *p = 0.014; *t* test. ***C***, Left, *Z*-projections of confocal image stacks of SACs stained for tdTomato at P7 (top) and P14 (bottom). Middle, Imaris reconstructions. Right, Magnified image of dendritic processes of SACs and Imaris reconstructions. Dotted circles indicate manually identified varicosities. ***D***, Number of manually identified varicosities per SAC during the second postnatal week. *n* = 5 SACs per age. Kruskal–Wallis rank sum *p < 0.01. Pairwise Wilcoxon rank sum tests revealed significant differences in varicosity count beginning at P10 (P10 vs P9, *p* = 0.048; P10 vs P8, *p* = 0.026; P10 vs P12, *p* = 0.015; P9 vs P8, *p* = 0.119; P12 vs P14, *p* = 0.690; false discovery rate corrected).

First, we used paired recordings to directly assay SAC→DSGC connectivity (Fig. 1*A*). As in Morrie and Feller, 2015, we sequentially patched onto multiple SACs surrounding a single DSGC, enabling us to construct spatial maps of overall inhibitory conductance. We found that while paired recordings at P9 revealed small inhibitory SAC→DSGC conductances and symmetric connectivity, inhibitory conductances at P10 increased for null side pairs, leading to a significant asymmetry in total inhibition (Fig. 1*B*). Thus, asymmetric connectivity is established by P10, although inhibitory conductances from null-oriented SACs to DSGCs are still smaller than those in adult animals (Wei et al., 2010).

Second, we utilized the unique morphology of the SAC to analyze formation of presynaptic structures during development. Specifically, during the first two postnatal weeks, SACs undergo a large morphological shift in their distal processes in which filopodial structures give way to thin processes with swellings, termed varicosities (Zheng et al., 2004; Lefebvre et al., 2012). Ultrastructural reconstructions of the direction-selective circuit have identified these swellings as presynaptic GABA release sites (Famiglietti, 1991; Briggman et al., 2011). To determine when these structures emerge on the SAC process during development, we sparsely labeled SACs using an inducible Chat::CreER × LSL-tdTomato mouse and low doses of tamoxifen. After amplifying the tdTomato signal via immunohistochemistry, high-resolution confocal imaging enabled precise reconstructions of SAC arbors throughout development (Fig. 1*C*). By counting varicosities on SACs from P7 to P14, we found that varicosities begin to emerge at P10, reaching mature levels by P12 (Fig. 1*D*). Thus, our morphological correlate of synaptogenesis corroborates our physiologically identified time point of P10 as the beginning of asymmetric SAC→DSGC synaptogenesis.

### Identification of synaptogenic factors that differentiate ventral- from nasal-preferring DSGCs

We hypothesize that asymmetric wiring between individual SAC processes and DSGCs is mediated in part by distinct synaptogenic molecules expressed on the dendrites of each subtype of DSGC and that these molecular differences are manifested at the transcriptional level at P10, when the asymmetric wiring emerges (Wei et al., 2010; Yonehara et al., 2010; Morrie and Feller, 2015).

**Figure 2.**
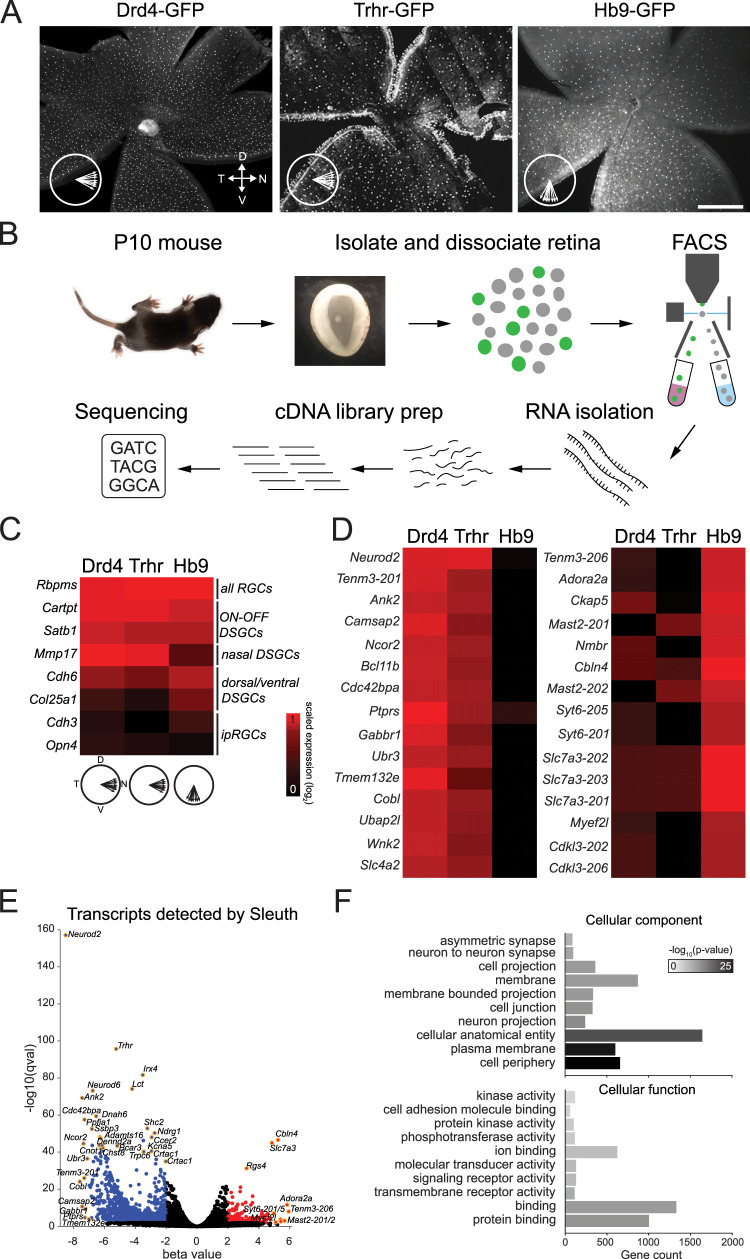
RNA sequencing strategy and identification of differentially expressed genes. ***A***, Representative fluorescence images of live whole-mount retinas showing the distribution of GFP+ DSGCs in the *Drd4-* (left), *Trhr*- (middle), and *Hb9-GFP* (right) BAC transgenic mouse lines. *Drd4-*GFP and *Trhr-GFP* images adapted from Huberman et al. (2009) and Rivlin-Etzion et al. (2011). Arrows indicate population tuning preferences among each DSGC type. Scale bar, 500 μm. ***B***, Sample collection and bulk RNA sequencing protocol. Retinas were isolated from P10 mice and then dissociated in papain. GFP+ cells were separated via FACS and processed for RNA isolation, cDNA library preparation, and RNA sequencing. P10 mouse image from The Jackson Laboratory (2012). ***C***, Validation of RNA-seq using known RGC or DSGC markers. Scaled expression is calculated as the log_2_ transformation of estimated counts for each gene. ***D***, Top 15 differentially expressed transcripts which were enriched in horizontal-preferring DSGCs (left) or vertical-preferring DSGCs (right). ***E***, Volcano plot of −log_10_(qval) versus β value (effect size) showing transcripts significantly upregulated (red) or downregulated (blue) in ventral-preferring DSGCs. *q* value cutoff was 0.01, and β cutoffs were less than or equal to −2 and ≥2. A subset of transcripts which passed these criteria are denoted with text and a gold outline around the corresponding point. ***F***, Top 10 GO terms for cellular component (top) and cellular function (bottom) for genes identified as significantly differentially expressed between nasal- and ventral-preferring DSGCs. Similar analysis was completed comparing differentially expressed transcripts between *Trhr-GFP* and Drd4-GFP DSGCs (Extended Data Fig. 2-1), Drd4-GFP and *Hb9-GFP* DSGCs (Extended Data Fig. 2-2), *Trhr-GFP* and *Hb9-GFP* DSGCs (Extended Data Fig. 2-3), and the intersection of transcripts that were differentially expressed between Drd4-GFP and *Hb9-GFP* DSGCs and between *Trhr-GFP* and *Hb9-GFP* DSGCs (Extended Data Fig. 2-4).

To identify these synaptogenic factors, we carried out the following RNA-seq-based screen. We isolated GFP-labeled cells in three different BAC-transgenic mouse lines that label two orthogonal preferred directions (Fig. 2*A*): Drd4-GFP (Huberman et al., 2009) and *Trhr-GFP* (Rivlin-Etzion et al., 2011), which label nasal-preferring DSGCs, and *Hb9-GFP* (Trenholm et al., 2011), which labels ventral-preferring DSGCs. We isolated GFP+ RGCs via FACS (Fig. 2*B*) and compared their transcriptional profiles to identify genes shared by nasal-preferring populations and genes that differentiate nasal- from ventral-preferring populations (Fig. 2*C–E*). This strategy was relatively straightforward for the *Drd4-GFP* line, in which nasal-preferring DSGCs are the only GFP+ cells in the retina. To isolate *Trhr-GFP* and *Hb9-GFP* DSGCs, however, we had to colabel DSGCs with tdTomato using the *VGlut2-Cre* mouse line to distinguish them from other GFP-expressing cells during FACS (see Materials and Methods).

10.1523/JNEUROSCI.1461-23.2024.f2-1 Figure 2-1 Download Figure 2-1, TIF file.

10.1523/JNEUROSCI.1461-23.2024.f2-2 Figure 2-2 Download Figure 2-2, TIF file.

**Figure 3.**
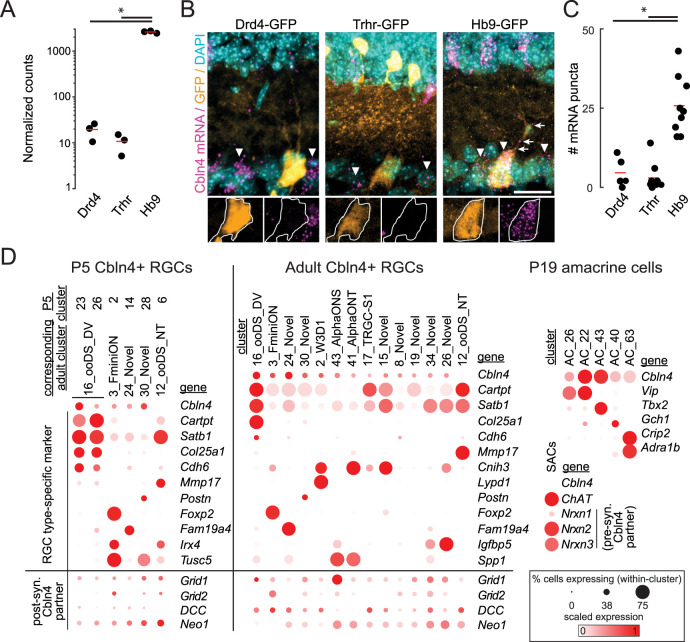
Cbln4 mRNA is enriched in ventral-preferring DSGCs and other RGC and amacrine cell subpopulations. ***A***, Estimated read counts generated from Kallisto quantification of *Cbln4* mRNA expression from bulk RNA-seq (Drd4-GFP, 19.96 ± 5.29; *Trhr-GFP*, 11.03 ± 3.33; *Hb9-GFP*, 2,654 ± 71.25; Wald test *q* value = 1.97 × 10^−47^. *n* = 3 biological replicates per genotype). ***B***, Top, FISH images showing *Cbln4* mRNA expression (magenta) in Drd4-GFP (left), *Trhr-GFP* (middle), and *Hb9-GFP* DSGCs (right) at P10. Arrowheads denote *Cbln4* mRNA-positive somas in the GCL. Arrows indicate *Cbln4* mRNA puncta colocalized with an *Hb9-GFP* dendrite in the IPL. Bottom, insets from top fluorescence images with GFP and *Cbln4* mRNA channels separated to highlight differential expression between GFP+ DSGCs, which are outlined in white. Scale bar, 20 μm. ***C***, Quantification of *Cbln4* mRNA expression at P10. Each dot denotes the number of fluorescent puncta for a given DSGC. One-way ANOVA *p* = 1.14 × 10^−6^. Two-sample *t* tests were performed for pairwise expression comparisons in ***C***; *p < 0.05 (Drd4-GFP, 4.6 ± 2.1 puncta/cell; *Trhr-GFP*, 3.0 ± 1.5 puncta/cell; *Hb9-GFP*, 25.8 ± 3.1 puncta/cell; *n*, Drd4-GFP = 5 cells/2 mice; *n*, *Trhr-GFP* = 9 cells/2 mice; *n*, *Hb9-GFP* = 9 cells/2 mice). ***D***, scRNA-seq expression data exported from the Broad Institute Single Cell Portal (Tran et al., 2019; Yan et al., 2020; Shekhar et al., 2022). Left and middle, Expression data for all P5 and adult RGC transcriptional clusters which express *Cbln4* mRNA in 10% or more cells. The rightmost cluster for these panels contains expression data for nasal/temporal (NT) ON–OFF DSGCs, which do not express detectable *Cbln4* mRNA. P5 clusters were matched to adult clusters via coexpression of cluster-defining markers at each age. Bottom left and middle panels show expression of postsynaptic markers which have been reported to interact with Cbln4 in some capacity. Right, Expression data for *Cbln4* in amacrine cell clusters. Also shown are expression levels of presynaptic neurexins in SACs. Scaling of expression is relative to each gene's expression across all cells in each cluster.

10.1523/JNEUROSCI.1461-23.2024.f2-3 Figure 2-3 Download Figure 2-3, TIF file.

**Figure 4.**
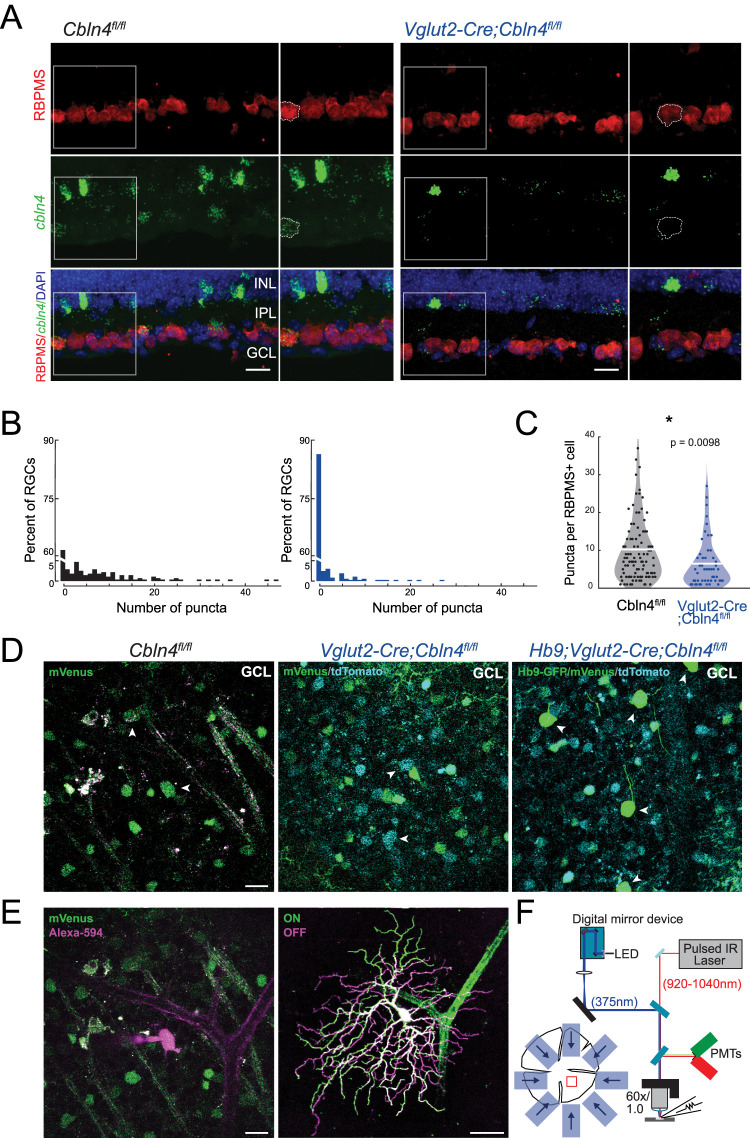
Cerebellin-4 reporter expression in *Cbln4*^fl/fl^ mice and *Vglut2-Cre;Cbln4*^*fl/fl*^ mice and approach for DSGC targeting. ***A***, Confocal images of *Cbln4*^fl/fl^ (left) and *Vglut2-Cre;Cbln4*^*fl/fl*^ (right) retina slices stained using RNAscope in situ hybridization using probes for *Cbln4* (green) and immunohistochemistry using RBPMS antibody to label RGCs (red). Panels on the right are magnifications of insets in the left panels for each condition. Scale bar, 20 µm. GCL, ganglion cell layer; IPL, inner plexiform layer; INL, inner nuclear layer. ***B***, Quantification of *Cbln4* expression as histograms showing the percent of RBPMS+ RGCs that express varying levels of *Cbln4* in Cbln4^fl/fl^ (black; 267 cells) and *Vglut2-Cre;Cbln4*^*fl/fl*^ (blue; 354 cells) retinas. ***C***, Quantification of *Cbln4* expression counted as punctate dots per RBPMS+ cell in Cbln4^fl/fl^ (black; 102 cells) and *Vglut2-Cre;Cbln4*^*fl/fl*^ (blue; 51 cells) retinas. Unpaired *t* test. ***D***, Representative two-photon fluorescence images of reporter expression in the GCL. Left, mVenus expression in Cbln4^fl/fl^ retina. Arrowheads denote putative Cbln4+ RGCs, the lower of which corresponds to the DSGC targeted in ***B***. Middle, tdTomato and mVenus expression in *VGlut2-Cre;Cbln4^fl/fl^* retina. Arrowheads denote tdTomato+ *Cbln4^−/−^* RGCs. Right, GFP, tdTomato, and mVenus expression in *Hb9-GFP;VGlut2-Cre;Cbln4^fl/fl^* retina. Arrowheads denote *Hb9-GFP+*, *Cbln4^−/−^* DSGCs. Scale bar, 20 μm. ***E***, Left, Fluorescence image of the same field of view as in ***A***, left, showing a targeted ventral-preferring DSGC cell body filled with Alexa-594 from a recording electrode. Scale bar, 20 μm. Right, Maximum intensity projection of a 3D fluorescence image of the dye-filled DSGC in ***B***, left. ON- (green) and OFF-stratifying (magenta) dendrites were segmented for further analysis. Scale bar, 50 μm. ***F***, Schematic of experimental setup. A two-photon microscope is fitted with an LED and digital mirror device for visual stimulation and a whole-cell recording electrode for voltage-clamp recording from DSGCs. Visually evoked calcium transients or synaptic currents were recorded during presentation of moving bars in eight directions.

10.1523/JNEUROSCI.1461-23.2024.f2-4 Figure 2-4 Download Figure 2-4, TIF file.

We performed differential expression analysis at the gene and transcript level, enabling identification of candidates down to the level of splice isoform. First, we validated expression of known DSGC markers on our data set [e.g., *Cdh6*, *Col25a1*, *Trhr*, and *Mmp17* (Morrie and Feller, 2016) and exclusion of non-DSGC markers (e.g., *Opn4*; Fig. 2*C*]. Next, we identified many other genes enriched in nasal- or ventral-preferring DSGCs at P10 (Fig. 2*D*,*E*). A total of 2,270 individual transcripts and 979 genes were identified by Kallisto/Sleuth as significantly up- or downregulated in ventral versus nasal DSGCs [adjusted *p* <0.01 and β value (effect size) less than −2 or >2]. GO analysis using top differentially expressed transcripts revealed enrichment of genes within the following cellular components: cell periphery (659 genes; *p* = 8.68 × 10^−25^), plasma membrane (602 genes; *p* = 1.40 × 10^−22^), neuron projection (242 genes; *p* = 1.38 × 10^−11^), cell junction (329 genes; *p* = 5.48 × 10^−11^), and neuron→neuron synapse (98 genes; *p* = 2.2 × 10^−9^). Top GO terms for molecular function included binding (1,333 genes; *p* = 4.4 × 10^−10^), transmembrane signaling receptor activity (113 genes; *p* = 8.08 × 10^−9^), molecular transducer activity (129 genes; *p* = 2.03 × 10^−7^), and cell adhesion molecule binding (59 genes; *p* = 4.55 × 10^−5^; Fig. 2*F*).

In a separate set of analyses, we performed additional pairwise differential expression tests between *Drd4-GFP* versus *Trhr-GFP* (Extended Data Fig. 2-1), *Drd4-GFP* versus *Hb9-GFP* (Extended Data Fig. 2-2), and *Trhr-GFP* versus *Hb9-GFP* (Extended Data Fig. 2-3). We then intersected the differentially expressed transcripts which passed our criteria for *Drd4-GFP* versus *Hb9-GFP* and *Trhr-GFP* versus *Hb9-GFP*. Comparing this approach with our earlier differential expression test of nasal- versus ventral-preferring DSGCs, there were 1,424 transcripts common to both sets, 846 transcripts unique to the nasal- versus ventral test, and 356 transcripts unique to the intersected pairwise differential expression set (Extended Data Fig. 2-4).

Our analysis of the nasal-preferring *Trhr-GFP* and *Drd4-GFP* DSGCs revealed interesting expression patterns which are reflective of their differences in developmental physiology and central projections (Rivlin-Etzion et al., 2011; Stafford et al., 2014). GO analysis using the top 500 differentially expressed genes (sorted by adjusted *p* value) revealed enrichment of the following cellular processes: neuron development (87 genes; *p* = 4.66 × 10^−19^), neurogenesis (109 genes; *p* = 6.41 × 10^−19^), nervous system development (137 genes, *p* = 2.78 × 10^−18^), neuron projection development (79 genes; *p* = 3.35 × 10^−18^), and neuron projection morphogenesis (61 genes; *p* = 6.31 × 10^−17^). In particular, G-protein-regulated inducer of neurite outgrowth 1 (Gprin1), which has been shown to influence axon outgrowth in other brain regions (Nordman and Kabbani, 2012; Nordman et al., 2014), was highly enriched in Drd4-GFP cells relative to *Trhr-GFP* and *Hb9-GFP* cells (Extended Data Fig. 2-3). Furthermore, several NMDA receptor (NMDAR) and NMDAR-associated genes were expressed by both nasal-preferring DSGC populations, consistent with their functional expression during development (Stafford et al., 2014). Notably, expression of the ifenprodil-sensitive subunits GluN1 and GluN2b (also known as Grin1 and Grin2b; Tajima et al., 2016) was present in both cells but significantly higher in Drd4-GFP despite functional evidence that this population loses ifenprodil sensitivity by adulthood in mice (Stafford et al., 2014).

To identify candidate molecules which may promote asymmetric SAC→DSGC wiring, we focused our selection to molecules which are (1) involved in synaptogenesis or cell→cell communication and (2) expressed at the cell surface or synapse, as determined by GO annotation or manual review of relevant literature. At the whole-gene level, numerous SAC→DSGC wiring candidates emerged as being differentially expressed between nasal- and ventral-preferring DSGCs at P10. These included members of the C1q/TNF superfamily and other complement-related factors (*Cbln4*, *C1qtnf1*, *C1qtnf6*, *C1ra*, *C1s1*), protein tyrosine phosphatases (*Ptprs*, *Ptpru*, *Ptprd*, *Ptprj*, *Ptprk*, *Ptprf*, *Ptprh*), and various cell adhesion molecules including members of the clustered protocadherin family (*Pcdhga4*, *Pcdhgb6*, *Pcdhgb2*, *Pcdhga10*, *Pcdhgb7*, *Pcdh1*, *Pcdhga3*, *Pcdhgb8*, *Pcdha6*, *Pcdh11x*). We highlight these families because of their critical role in synapse formation or remodeling in other brain circuits (Dunah et al., 2005; Stevens et al., 2007; Matsuda, 2017; Yuzaki, 2018; Han et al., 2020; Sanes and Zipursky, 2020). Note, a recent study using single-cell RNA-seq to differentiate subtypes of ON-DSGCs also identified *Ptprk* and additional factors as possible regulators of direction-selective circuit development (Al-Khindi et al., 2022).

Our bulk RNA sequencing approach provided a read depth that enabled identification of differentially expressed genes at the splice isoform level, which are known to contribute to synapse specificity throughout the brain (Furlanis and Scheiffele, 2018). For example, differential splicing of teneurins has been implicated in wiring in the hippocampus (Berns et al., 2018; Li et al., 2020). Interestingly, we observed two distinct splice variants of the homophilic cell adhesion molecule Tenm3 which were differentially expressed between nasal- and ventral-preferring DSGCs, with Tenm3-201 enriched in nasal DSGCs and Tenm3-206 enriched in ventral DSGCs (Fig. 2*D*,*E*). Although we did not further explore the role of differential splicing in SAC→DSGC asymmetric synaptogenesis, we discuss the role of Tenm3 in visual system development below.

For this study, we elected to further study the synaptic organizer Cbln4. *Cbln4* was a top differentially expressed transcript in our analysis, showing >100-fold enrichment in ventral-preferring *Hb9-*DSGCs (Fig. 3*A*). *Cbln4* is one isoform of the C1q family of secreted transsynaptic glycoproteins. It is expressed in subpopulations of neurons throughout the brain, mediates cell type-specific synaptogenesis or circuit-specific functions across brain regions, and has been implicated in specifying wiring of subtypes of inhibitory interneurons onto cortical pyramidal cells (Seigneur and Südhof, 2017; Seigneur et al., 2018; Favuzzi et al., 2019; Liakath-Ali et al., 2022). In the retina, however, the function of cerebellins during circuit formation is unknown.

### Cerebellin-4 is preferentially expressed in ventral-preferring DSGCs and subsets of other retinal neurons

Using fluorescence in situ hybridization (FISH), we verified that *Cbln4* mRNA was present in ventral-preferring *Hb9-GFP* cells and absent in nasal-preferring Drd4-GFP and *Trhr-GFP* cells (Fig. 3*B*,*C*). FISH also revealed *Cbln4* expression in several other cell types in both the GCL and inner nuclear layer (INL), with relatively high expression in a subset of cells in the INL (Fig. 3*B*). We often observed *Cbln4* mRNA expression in the IPL outside of DAPI-stained cell bodies, highlighting the potential for local translation of Cbln4 in RGC dendrites (Holt et al., 2019).

These FISH data are consistent with scRNA-seq datasets which show that *Cbln4* mRNA is enriched ventral-/dorsal-preferring DSGCs and absent in nasal/temporal DSGCs. *Cbln4* mRNA is also present in a subset of amacrine cells, particularly those expressing VIP, Tbx2, or Crip2 (Yan et al., 2020), including VIP amacrine cells that are in the GCL (Akrouh and Kerschensteiner, 2015; Park et al., 2015). scRNA-seq has also revealed *Cbln4* expression in non-direction-selective RGCs including F-mini-ON RGCs and ON-alpha RGCs. Furthermore, the pre- and postsynaptic binding partners of Cbln4 are expressed by amacrine cells and RGCs. Namely, Nrxn1-3 are highly expressed across the amacrine cell populations, including SACs, while DCC, Neo1, and GluD1 are expressed postsynaptically in dorsal/ventral DSCGs and other RGC subpopulations (Fig. 3*D*; Tran et al., 2019; Goetz et al., 2022; Jacobi et al., 2022; Shekhar et al., 2022). Hence, while Cbln4 could potentially influence synapse specificity in any of these neuronal types, the strong differences in its expression between nasal- and ventral-preferring DSGCs compelled us to test its role in asymmetric SAC→DSGC wiring.

### RGC-targeted knock-out of Cbln4 does not strongly impact tuning of ventral DSGCs

To test for a role of Cbln4 in asymmetric SAC→DSGC synaptogenesis, we generated an RGC-specific knock-out by crossing a reporter-conditional knock-out mouse strain, *Cbln4^fl/fl^* (Seigneur and Südhof, 2017; Seigneur et al., 2018) with a *VGlut2-Cre* mouse line, which expresses Cre in nearly all RGCs (*Vglut2-Cre;Cbln4^fl/fl^*; Ellis et al., 2016). In the absence of Cre, Cbln4-positive cells in *Cbln4^fl/fl^* mice express functional Cbln4 and mVenus. In the presence of Cre, *Cbln4^fl/fl^* cells have no functional Cbln4 and express tdTomato. FISH revealed significant *Cbln4* mRNA knockdown in *Vglut2-Cre;Cbln4^fl/fl^* retinas (Fig. 4*A–C*). Fluorescent reporter expression in *Cbln4^fl/fl^* was consistent with *Cbln4* expression patterns observed using FISH and scRNA-seq (Fig. 4*D*). Expression of mVenus or tdTomato was used to target *Cbln4^fl/fl^* or *Vglut2-Cre;Cbln4^fl/fl^* RGCs for whole-cell recording and morphological assessment (Fig. 4*E*,*F*). In a subset of experiments, we crossed these mice with *Hb9-GFP* mice to more robustly label ventral DSGCs for targeted recordings (Fig. 4*D*, right). Note, *Hb9-GFP* cells represent a subset of all ventral preferring DSGCs (Tiriac et al., 2022).

**Figure 5.**
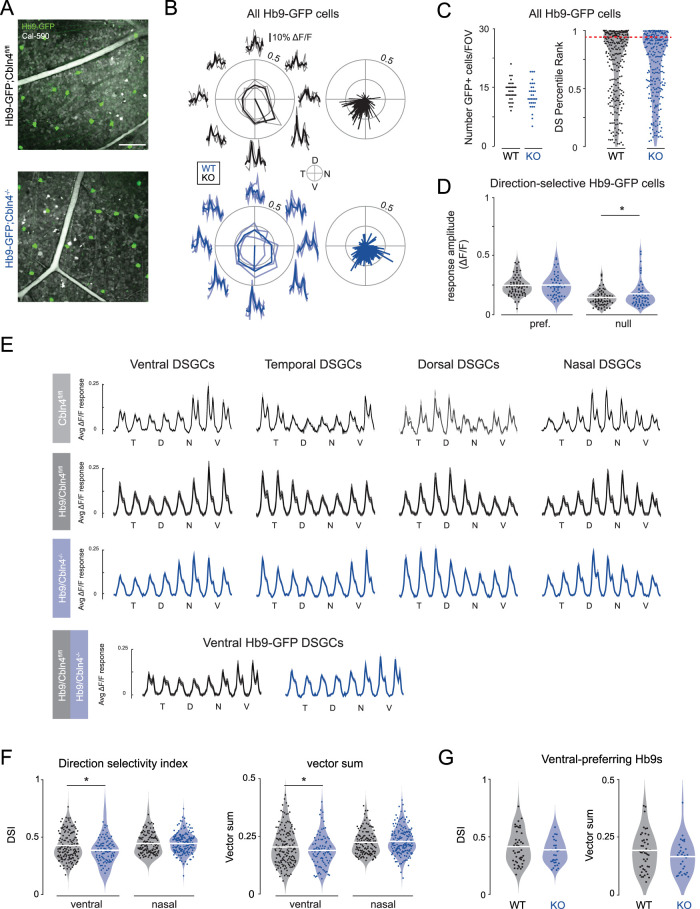
Population calcium imaging of *Cbln4^fl/fl^* and *Vglut2-Cre;Cbln4^fl/fl^* DSGCs during moving bar stimuli. ***A***, Two example fields of view (FOVs) showing *Hb9-GFP* DSGCs (ventral-preferring) in *Cbln4^fl/fl^* (top) and *Vglut2-Cre;Cbln4*^*fl/fl*^ (which we refer to as Cbln4^−/−^, bottom). Scale bar, 100 µm. ***B***, Left, Example tuning curves for individual *Hb9-GFP* DSGCs with calcium responses corresponding to each of the eight presented directions. Right, Polar plots of *Hb9-GFP* population tuning from Cbln4^fl/fl^ and *Vglut2-Cre;Cbln4^fl/fl^* mice, where each line represents a cell's preferred direction and vector sum. *n*, *Cbln4^fl/fl^*, 265 *Hb9-GFP* DSGCs/5 mice; *n*, *Vglut2-Cre;Cbln4^fl/fl^*, 252 Hb9-DSGCs/4 mice. ***C***, Left, Average number of *Hb9-GFP* cells per FOV in Cbln4^fl/fl^ and *Vglut2-Cre;Cbln4*^*fl/fl*^ mice. *p* = 0.88, unpaired *t* test. Right, Summary data for the percentile rank of each cell's DSI compared with permutations where the directions of the moving bar are block shuffled. For reference, 95th percentile is considered statistically significantly direction selective (red dashed line; *p* = 0.53; unpaired *t* test). ***D***, Summary data of max Δ*F* / *F* response to moving bars among significantly direction selective *Hb9-GFP* cells in the preferred and null direction in *Cbln4^fl/fl^* and *Vglut2-Cre;Cbln4^fl/fl^* mice. *n*, *Cbln4^fl/fl^*, 73 *Hb9-GFP* DSGCs/5 WT mice; *n*, Cbln4^−/−^, 57 Hb9-DSGCs/4 KO mice. For all panels, data corresponding to *Cbln4^fl/fl^* (WT) are black, and those corresponding to *Vglut2-Cre;Cbln4^fl/fl^* (KO) are blue. *p < 0.05, unpaired *t* test. ***E***, Average Δ*F* / *F* response for moving bars in eight different directions for all significantly direction-selective RGCs for each preferred direction. ***F***, DSI and vector sum of significantly direction-selective ventral-preferring ganglion cells compared with those of significantly direction-selective nasal-preferring cells in *Cbln4^fl/fl^* and *Vglut2-Cre;Cbln4*^*fl/fl*^ mice. *n*, *Cbln4^fl/fl^*, 163 ventral-preferring, 171 nasal-preferring/5 mice; *n*, *Vglut2-Cre;Cbln4*^*fl/fl*^, 96 ventral-preferring, 133 nasal-preferring/4 KO mice. *p < 0.05, unpaired *t* test. ***G***, Same as ***F*** but for only *Hb9-GFP* DSGCs (ventral-preferring). *n*, *Cbln4^fl/fl^*, 48 *Hb9-GFP* DSGCs/5 mice; *n*, *Vglut2-Cre;Cbln4^fl/fl^*, 39 *Vglut2-Cre;Cbln4^fl/fl^*; Hb9 mice. See Table 1 for description of populations.

We performed population two-photon calcium imaging to compare the tuning properties of nasal- and ventral-preferring DSGCs in response to moving bar stimuli, utilizing the *Cbln4^fl/fl^*;*Hb9-GFP* and *Vglut2-Cre;Cbln4^fl/fl^*;*Hb9-GFP* mouse lines (Fig. 5*A*). To quantify directional tuning, we calculated the vector sum and DSI of peak calcium responses across all stimulus directions for *Hb9-GFP*+ cells and other DSGCs. In both *Cbln4^fl/fl^* and *Vglut2-Cre;Cbln4^fl/fl^* retinas, the population of Hb9-GFP+ cells remained ventrally tuned (Fig. 5*B*). We performed a permutation test to identify significantly direction-selective cells (see Materials and Methods; Table 1) and found no significant difference in the number of Hb9-positive cells that were direction selective (Fig. 5*C*). For preferred direction stimuli, we found no change in response amplitude of direction-selective *Hb9-GFP* cells; however, for null direction stimuli, we found a small but significant increase in the maximum amplitude in *Vglut2-Cre;Cbln4^fl/fl^* retinas (Fig. 5*D*).

To characterize the tuning of the entire ventral-preferring DSGC population, including *Hb9-GFP* cells, we performed a *k*-means analysis to cluster DSGCs by preferred direction (see Materials and Methods). Robust responses were found for all four subtypes of ON–OFF DSGCs (Fig. 5*E*). In *Vglut2-Cre;Cbln4^fl/fl^* retinas, ventral-preferring DSGCs had a significant reduction in their tuning, as quantified by the DSI and vector sum of their calcium responses. There was no significant difference among nasal DSGCs between the two genotypes (Fig. 5*F*). These data indicate that in the *Vglut2-Cre;Cbln4^fl/fl^* mice, ventral DSGCs have reduced tuning due to increased response for null stimulation, consistent with an impairment in inhibitory synapse formation.

However, when we restricted the analysis to ventral-preferring DSGCs that were Hb9-positive, there was no significant difference in DSI or vector sum in *Hb9-GFP;Cbln4^fl/fl^* or *Hb9-GFP*;*Vglut2-Cre;Cbln4^fl/fl^* retinas (Fig. 5*G*; note *Hb9-GFP* cells comprised 28% of all ventral-preferring DSGCs in *Cbln4^fl/fl^* and 30% of all ventral-preferring DSGCs in *Vglut2-Cre;Cbln4^fl/fl^*; Table 1). When we repeated this analysis on all *Hb9-GFP* neurons, independent of their tuning, we found no differences in response amplitude or directional tuning (data not shown). Hence, the small reduction in tuning among all ventral-preferring DSGCs in the RGC-*Cbln4^−/−^* retinas was not observed in the subset of ventral-preferring DSGCs that are labeled in *Hb9-GFP* retinas.

An important caveat to these findings is that Hb9-GFP+ neurons have a component of their direction selectivity that is independent of asymmetric inhibition and is attributed to their asymmetric dendrites (Trenholm et al., 2011). Importantly, we have not observed GABA-independent tuning of DSGCs using two-photon population calcium imaging (Bos et al., 2016), likely due to the reduced signal-to-noise relative to cell-attached recordings.

### Directionally tuned inhibition is retained in *Cbln4^−/−^* DSGCs

**Figure 6.**
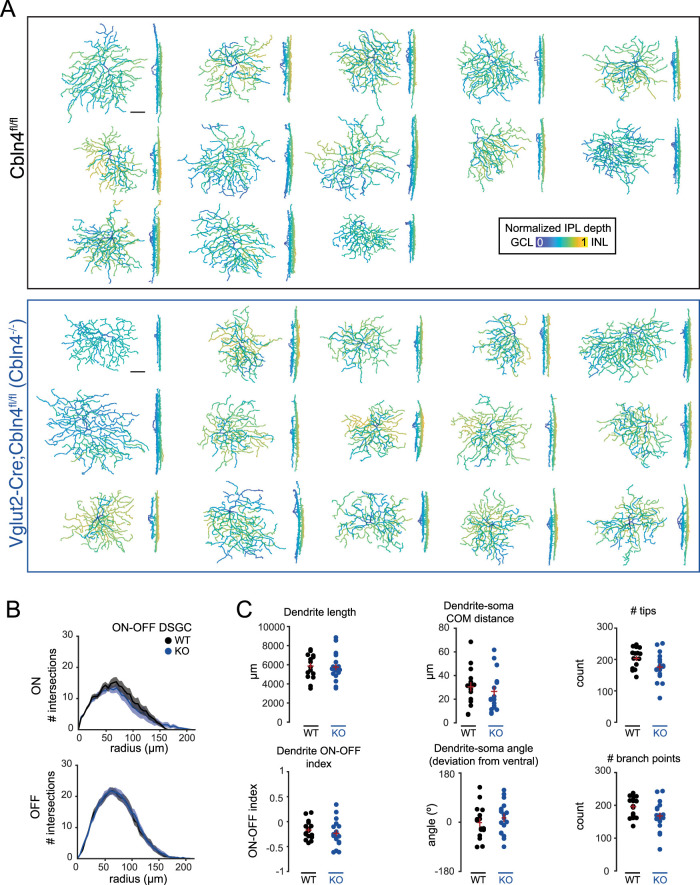
Dendritic morphology of ventral-preferring ON–OFF DSGCs is preserved in *Cbln4^−/−^* retinas. ***A***, Maximum intensity projections of ventral-preferring ON–OFF DSGC reconstructed dendritic skeletons, color coded by IPL depth, in *Cbln4^fl/fl^* (top) and *Vglut2-Cre;Cbln4^fl/fl^* (bottom) mice. Scale bar, 50 µm. ***B***, Sholl intersection profiles for ventral-preferring ON–OFF DSGCs. Top, ON-dendritic arbor. Bottom, OFF-dendritic arbor. ***C***, Summary data for dendritic morphology quantification of ventral-preferring DSGCs from *Cbln4^fl/fl^* and *Vglut2-Cre;Cbln4^fl/fl^* mice. Top, from left to right, Total dendrite length, dendrite–soma center-on-mass (COM) distance, and number of dendritic tips. Bottom, from left to right, Dendrite ON–OFF index, dendrite–soma angle deviation from ventral direction, and number of branch points. Error bars represent SEM. *n*, *Cbln4^fl/fl^*, 16 ventral-preferring DSGCs/10 mice; *n*, *Vglut2-Cre;Cbln4^fl/fl^*, 18 ventral-preferring DSGCs/8 mice.

**Figure 7.**
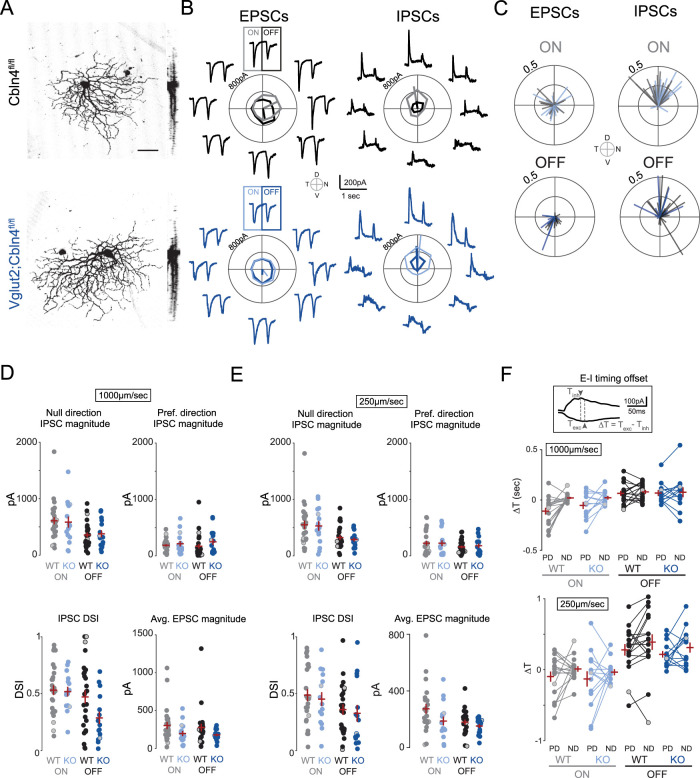
Tuning, strength, and timing of synaptic inputs onto ventral-preferring ON–OFF DSGCs during moving bar stimuli are preserved in *Vglut2-Cre;Cbln4^fl/fl^* retinas. ***A***, Maximum intensity projections of volumetric two-photon images of dye-filled ventral-preferring ON–OFF DSGCs in *Cbln4^fl/fl^* (top) and *Vglut2-Cre;Cbln4^fl/fl^* (bottom) mice. Scale bar, 50 µm. ***B***, Example EPSCs (left) and IPSCs (right) recorded from *Cbln4^fl/fl^* (top) and *Vglut2-Cre;Cbln4^fl/fl^* (bottom) ventral-preferring DSGCs during drifting bar stimuli at 1,000 µm/s. Recordings correspond to cells in ***A***. Polar plots in middle of current traces show peak onset (gray or light blue) and offset (black or dark blue) current for each direction, along with vector sum magnitudes and directions. ***C***, Population polar plots showing tuning of EPSCs (left) and IPSCs (right) among ventral-preferring DSGCs from *Cbln4^fl/fl^* and *Vglut2-Cre;Cbln4^fl/fl^* mice, where each line represents a cell's preferred direction and vector sum for EPSCs or IPSCs. *n*, *Cbln4^fl/fl^*: 35 ventral-preferring DSGCs/10 mice; *n*, *Vglut2-Cre;Cbln4^fl/fl^*: 17 ventral-preferring DSGCs/8 mice. ***D***, Summary data showing all ventral-preferring DSGC synaptic responses to stimuli moving at 1,000 µm/s. IPSC magnitude in the null direction (top left), IPSC magnitude in the preferred direction (top right), IPSC DSI (bottom left), and average EPSC magnitude (bottom right) for both genotypes and for ON and OFF responses. Preferred and null directions for each DSGC were determined using the vector sum angle for IPSCs. Cells that did not have asymmetric dendrites (based on the asymmetry index) are denoted with gray-filled circles. ***E***, Same for as ***D*** for stimuli moving at 250 µm/s. ***F***, Timing offsets between peak EPSCs and IPSCs in the preferred and null directions for both genotypes for bars moving at 1,000 µm/s (top) and bars moving at 250 µm/s (bottom). These analyses included cells that did not have asymmetric dendrites (gray-filled circles). All error bars represent SEM.

To directly assess the impact of Cbln4 on inhibitory synapse formation, we acquired whole-cell voltage-clamp recordings of excitatory and inhibitory postsynaptic currents (E/IPSCs) from *Cbln4^fl/fl^* or *Vglut2-Cre;Cbln4^fl/fl^* RGCs responding to moving bar stimuli. To take into account velocity tuning of synaptic contributions (Sivyer et al., 2019; Summers and Feller, 2022; Mani et al., 2023), we compared their responses at two different stimulus speeds. Each recorded RGC was filled with a morphological dye to enable 3D reconstruction and cell type verification. Cells which exhibited a distinctive bistratified morphology and well-defined postsynaptic responses to light onset and offset were classified as ON–OFF RGCs. These were further classified as DSGCs if they exhibited asymmetric inhibitory currents (DSI ≥ 0.2). We found no differences in the stratification, branching properties, or asymmetry between *Cbln4^fl/fl^* and *Vglut2-Cre;Cbln4^fl/fl^* ventral-preferring DSGC dendritic arbors (Figs. 6*A–C*, 7*A*).

We further classified these cells as ventral-preferring DSGCs if they exhibited dorsally tuned inhibitory currents and/or ventrally oriented dendrites. Using these criteria, we found similar proportions of ON–OFF DSGCs in *Cbln4^fl/fl^* and *Vglut2-Cre;Cbln4^fl/fl^* (*Cbln4^fl/fl^*, 25 DSGCs out of 65 mVenus+ *Vglut2-Cre;Cbln4^fl/fl^*, 25 DSGCs out of 68 tdTomato+ RGCs). A subset of recordings was carried out in *Cbln4^fl/fl^*;*Hb9-GFP* and *Vglut2-Cre;Cbln4^fl/fl^*;*Hb9-GFP* mice to verify this classification.

In both *Cbln4^fl/fl^* and *Vglut2-Cre;Cbln4^fl/fl^* retinas, ventral-preferring DSGCs exhibited stronger inhibition in response to bars moving in the dorsal direction, a hallmark feature of ventral-preferring DSGCs (Fig. 7*B*,*C*). We also observed directional excitation among many DSGCs during moving bar stimuli, with larger EPSCs in the ventral direction on average (Fig. 7*B*,*C*), though this tuning was much weaker than for IPSCs, consistent with previous reports (Park et al., 2014; Pei et al., 2015; Percival et al., 2019; El-Quessny et al., 2020; Summers et al., 2021). We observed no significant differences between genotypes in strength (Fig. 7*D*,*E*), tuning (Fig. 7*D*,*E*), or timing of excitatory or inhibitory synaptic inputs (Fig. 7*F*). These results persisted when we performed the analysis using only the subset of DSGCs that exhibited asymmetric dendrites (asymmetry index, >0.25; Fig. 7*D*,*E*) and for both faster bars (1,000 µm/s) and slower bars (250 µm/s). We also found no differences using other measures of tuning including total charge transfer, excitation:inhibition ratio, and speed tuning during moving bar stimuli (data not shown). Together, these results suggest that RGC-derived Cbln4 is not required for asymmetric inhibitory synaptogenesis between SAC processes and DSGCs.

### Morphology of and synaptic inputs onto *Cbln4^−/−^* non-DS RGCs

In addition to ON–OFF DSGCs, Cbln4 is expressed in a subset of other RGC types (Figs. 3*D*, 4*A*). To assess the impact of Cbln4 more broadly on the development of these other RGC types, we targeted all reporter-positive somas in the GCL in both *Cbln4^fl/fl^* and *Vglut2-Cre;Cbln4^fl/fl^* retinas for voltage-clamp recording and volumetric reconstructions of morphology. We confirmed that highly fluorescent mVenus+ cells with small somata in the GCL were primarily amacrine cells based on morphology and lack of axons, and these were excluded from this analysis. Notably, we never encountered tdTomato+ amacrine cells in *Vglut2-Cre;Cbln4^fl/fl^* retinas, highlighting the specificity of Cre expression among RGCs in the *Vglut2-Cre* mouse line.

**Figure 8.**
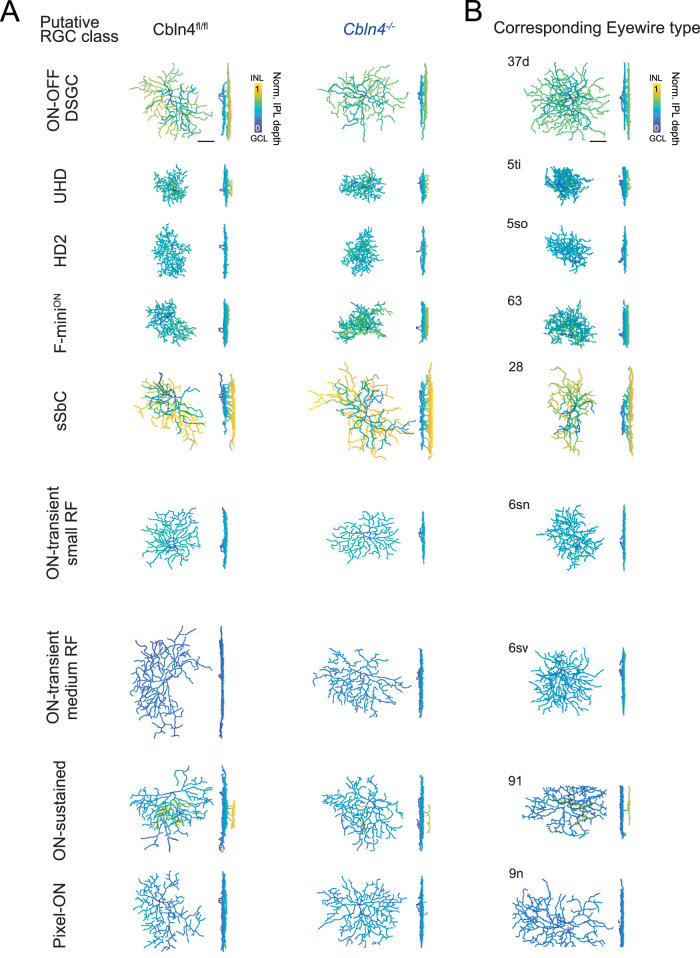
Cbln4 reporter and knock-out RGCs qualitatively align with defined morphological types. ***A***, Dendritic reconstructions of the most commonly encountered Cbln4-expressing RGC types are plotted and colored by normalized IPL depth for *Cbln4^fl/fl^* (left) and *VGlut2-Cre;Cbln4^fl/fl^* (right). ***B***, Cbln4-expressing RGC types were qualitatively matched to Eyewire Museum RGC types by their stratification profiles, arbor size, and dendritic branching characteristics. Scale bar, 50 μm. Dendritic reconstructions are also provided for Cbln4 reporter-expressing ON–OFF DSGCs (Extended Data Fig. 8-1), small receptive field ON–OFF RGCs (Extended Data Fig. 8-2), suppressed-by-contrast and other ON–OFF RGCs (Extended Data Fig. 8-3), and other unclassified RGCs (Extended Data Fig. 8-4).

**Figure 9.**
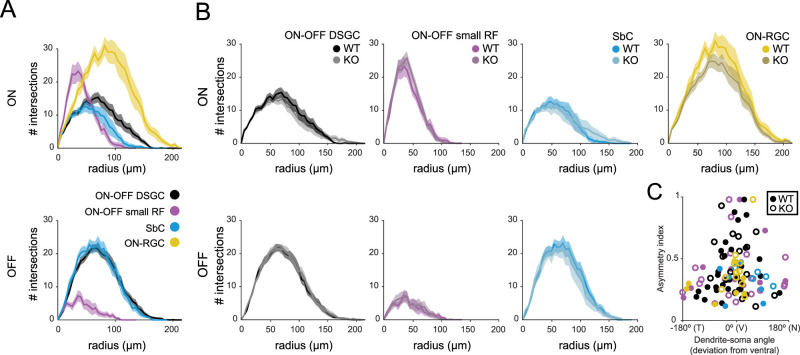
Cbln4 reporter-expressing RGCs exhibit distinct but overlapping morphological properties and ventrally oriented dendrites. ***A***, Sholl intersection profiles for the major Cbln4-positive RGC types identified. Top, ON-dendritic arbor. Bottom, OFF-dendritic arbor. ***B***, Sholl intersection profiles for the same cell types in ***A***, compared with type-matched *Vglut2-Cre;Cbln4^fl/fl^* RGCs. ***C***, Dendritic asymmetry index versus angle of asymmetry, calculated as deviation of dendrite–soma vector from the ventral orientation.

Recorded RGCs were broadly classified by their dendritic stratification profiles across the IPL. Dendrites of mVenus+ RGCs in *Cbln4^fl/fl^* retina were primarily bistratified (54/65 *Cbln4+* RGCs, including DSGCs) with a smaller subset that were monostratified in the ON (8/65 of *Cbln4+* RGCs) or OFF (3/65 *Cbln4+* RGCs) sublayers. Similar proportions were observed in tdTomato+ RGCs in *Vglut2-Cre;Cbln4^fl/fl^* retina (bistratified, 50/68; monostratified, 17/68 ON and 1/68 OFF). Within these broad stratification groups, we identified several distinct subtypes of RGCs which exhibited similar morphologies between *Cbln4^fl/fl^* and *Vglut2-Cre;Cbln4^fl/fl^* retinas (Fig. 8*A*) with no significant differences between genotypes as measured by a Sholl analysis (Fig. 9). Leveraging published datasets which combine RGC transcriptional profiling, morphology, and electrophysiology, we were able to match most of our recorded RGCs with established RGC types (Fig. 8*B*; Extended Data Figs. 8-1 to 8-4; Bae et al., 2018; Goetz et al., 2022; Huang et al., 2022; Jacobi et al., 2022).

10.1523/JNEUROSCI.1461-23.2024.f8-1 Figure 8-1 **Reconstructed morphologies of Cbln4 reporter-expressing ON-OFF DSGCs**. Color coded by IPL depth. Scale bars 50μm. Download Figure 8-1, TIF file.

10.1523/JNEUROSCI.1461-23.2024.f8-2 Figure 8-2 **Reconstructed morphologies of Cbln4 reporter-expressing small receptive field ON-OFF RGCs**. Cbln4^-/-^ = Vglut2-Cre;*Cbln4^fl/fl^*. Color coded by IPL depth. Scale bars 50μm. Download Figure 8-2, TIF file.

10.1523/JNEUROSCI.1461-23.2024.f8-3 Figure 8-3 **Reconstructed morphologies of Cbln4 reporter-expressing suppressed-by-contrast and other ON-OFF RGC**. Color coded by IPL depth. Scale bars: 50μm. Download Figure 8-3, TIF file.

10.1523/JNEUROSCI.1461-23.2024.f8-4 Figure 8-4 **Reconstructed morphologies of Cbln4 reporter-expressing ON, OFF, and one unclassified RGC.** Cbln4^-/-^ = Vglut2-Cre;*Cbln4^fl/fl^*. Color coded by IPL depth. Scale bars: 50μm. Download Figure 8-4, TIF file.

**Figure 10.**
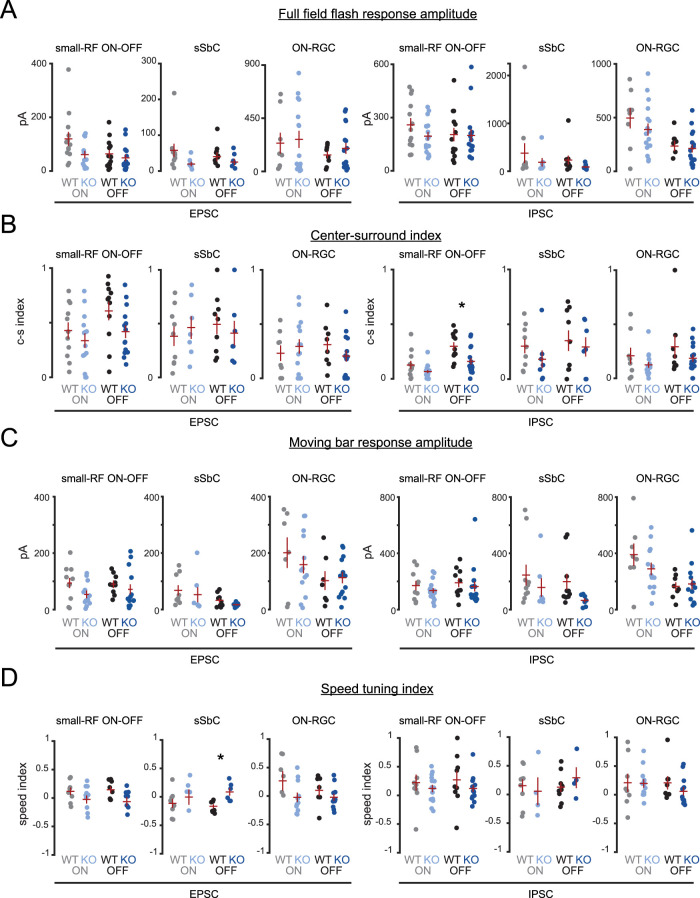
Strength and tuning of synaptic inputs in Cbln4 reporter-expressing non-direction-selective RGCs are preserved in *Vglut2-Cre;Cbln4^fl/fl^* retinas. ***A***, Peak ON- and OFF-EPSCs (left) and ON- and OFF-IPSCs (right) for *Cbln4^fl/fl^* (WT) and *Vglut2-Cre;Cbln4*^*fl/fl*^ (KO) mice during full-field flash stimuli. Responses are plotted separately for all small-receptive field (RF) ON–OFF, sustained suppressed-by-contrast (sSbC), and ON-RGCs. ***B***, Center-surround indices during stimulation with variable size spots, plotted as in ***A***. ***C***, Peak ON- and OFF-EPSCs (left) and ON- and OFF-IPSCs (right) during moving bar stimuli, plotted as in ***A*** and ***B***. ***D***, Speed tuning indices for the same cells as in ***A–C***, calculated from peak current responses to moving bars at 250 µm/s and 1,000 µm/s. All error bars represent SEM. *p < 0.05, unpaired *t* test.

The largest group of *Cbln4+* cells we characterized outside of ON–OFF DSGCs were ON–OFF small receptive field RGCs, including F-mini-ON and ultrahigh definition (UHD) RGCs. We also recorded from sustained suppressed-by-contrast and ON-transient alphas. To compare synaptic inputs among these cell types between *Cbln4^fl/fl^* and *Vglut2-Cre;Cbln4^fl/fl^* retinas, we recorded IPSCs and EPSCs in response to an array of visual stimuli, including spot flashes of light at varying diameter and drifting bars of different speeds. We observed no significant differences in peak EPSCs or IPSCs during stimulus onset or offset for ON–OFF small receptive field, suppressed-by-contrast, and ON-RGCs (Fig. 10*A*). Center-surround properties were also generally preserved, except for small receptive field ON–OFF RGCs, which had significantly reduced center surround indices for inhibition in *Vglut2-Cre;Cbln4^fl/fl^* (Fig. 10*B*). As with our findings in DSGCs, moving bar-evoked EPSCs and IPSCs were largely unchanged among *Vglut2-Cre;Cbln4^fl/fl^* small receptive field ON–OFF, suppressed-by-contrast, and ON-RGCs (Fig. 10*C*). Speed tuning indices across *Vglut2-Cre;Cbln4^fl/fl^* RGCs were largely unchanged as well, with the exception of suppressed-by-contrast RGCs, which exhibited a small but significant increase in speed tuning in the OFF pathway (Fig. 10*D*). Hence, RGC-derived Cbln4 impacted some synaptic circuits, but elucidating the details of these deficits requires further studies.

## Discussion

Here, we performed mRNA transcriptome analysis on three populations of direction-selective ganglion cells—two preferring horizontal motion and one preferring vertical motion—to identify differentially expressed genes which may promote asymmetric inhibitory synaptogenesis in the direction-selective circuit. Using this approach, we identified a variety of candidate molecules potentially involved in cell subtype-specific synaptogenesis within this circuit. We tested the role of one differentially expressed candidate, Cbln4, which was enriched in ventral-preferring DSGCs relative to nasal-preferring DSGCs. Using a targeted knock-out approach, we found that among ventral-preferring DSGCs, deletion of Cbln4 led to a small reduction in direction-selective tuning while maintaining dendritic morphology and normal strength and asymmetry of inhibitory synaptic transmission. Similarly, other Cbln4-expressing RGCs retained overall strength of inhibitory and excitatory responses to light in the absence of Cbln4. Perturbations of circuits that mediate center-surround organization and speed tuning among small receptive field ON–OFF and suppressed-by-contrast RGCs, respectively, suggest that RGC-derived Cbln4 provides minor circuit-specific contributions to visually evoked synaptic inputs. Overall, we have shown that this approach can be used to identify interesting candidate molecules, and future functional studies are required to reveal the mechanisms by which these candidates influence synaptic wiring within specific circuits.

It is difficult to know what underlies the small difference in tuning observed across all ventral preferring DSGCs in the *Vglut2-Cre;Cbln4^fl/fl^* given that there was no significant difference in tuning in the subset of ventral preferring DSGCs labeled by *Hb9-GFP*. This difference could be attributed to the variance of both measures and the fact that DSI compares measurements within individual cells and while response amplitude is compared across all cells.

### Factors influencing the development of direction-selective circuits

There has been tremendous progress in understanding the various steps in the development of direction-selective circuits including the identification of axon guidance and cell adhesion molecules that mediate the lamination of requisite cell types (reviewed in Hamilton et al., 2021). By P4, SACs form weak but symmetric inputs onto DSGCs (Wei et al., 2010). Here, we have focused on the subsequent step—the rapid increase in asymmetric inhibitory inputs from individual SAC processes with “appropriate” DSGC subtypes, which means each SAC process is oriented parallel to the null directions of the DSGCs with which it is connected (Wei et al., 2010; Yonehara et al., 2010). This maturation of asymmetric inhibition occurs during a short developmental period—P9–P11—and is mediated by an increase in synapse number rather than strength (Morrie and Feller, 2015). Hence, this 2-d developmental window represents a period of robust but highly precise synaptogenesis between SACs and DSGCs.

Our goal was to identify the postsynaptic molecules that contribute to this process of synapse specificity. The assumption is that the different subtypes of DSGCs, which are known to be unique cell types, express unique sets of synaptogenic factors that interact with asymmetrically localized factors on SAC processes. Our RNA-seq strategy revealed a broad set of transcripts encoding synaptic and cell surface molecules which were differentially expressed by horizontal- and vertical-preferring DSGCs. Many of these are members of well-characterized families of synaptic organizers and cell adhesion molecules, and as such are promising candidate factors for regulating SAC→DSGC wiring. First, we noted several differentially expressed genes in the clustered protocadherin family, whose combinatorial expression generates a molecular code which enables neurite self-recognition and self-avoidance across sensory systems (Lefebvre et al., 2012; Zhang et al., 2017; Mountoufaris et al., 2017; Meltzer et al., 2023). The *Pcdh-gamma* (*Pcdhg*) locus in particular has been implicated in SAC process self-avoidance, and mice with SAC-targeted *Pcdhg* mutations exhibit degraded retinal direction selectivity, possibly resulting from reduced SAC→SAC inhibition (Kostadinov and Sanes, 2015). The cell-autonomous function of DSGC-expressed *Pcdhg* in direction-selective circuit wiring, however, remains to be determined. Another class of molecules that was differentially expressed includes those in the C1q family of complement-related factors, which promote synapse reorganization via interactions with microglia and astrocytes (Stephan et al., 2012), or synapse stabilization via transsynaptic signaling (Matsuda et al., 2016; Matsuda, 2017; Zhong et al., 2017).

As noted in the results, the read-depth afforded by bulk RNA sequencing led to identification of different splice isoforms, including distinct Tenm3 splice isoforms enriched in different DSGCs. Teneurins are transmembrane proteins that have been implicated in several steps of neural circuit assembly including axon targeting, synaptogenesis, and synapse-specific wiring. There are four members of the family (Tenm1–4), which mediate transcellular interactions via both homophilic and heterophilic binding (for review, see Leamey and Sawatari, 2019). Tenm3 is highly expressed in the ventral retina (Leamey et al., 2007; Cheung et al., 2019), where it has been implicated in proper wiring of ipsilateral retinofugal projections (Dharmaratne et al., 2012; Merlin et al., 2013). Tenm3 has primarily been implicated in excitatory synapse formation, with evidence that it is localized presynaptically (Zhang et al., 2022). However, recently it was shown that in zebrafish retina, Tenm3 is expressed in inhibitory amacrine cells and is critical for proper synapse formation of circuits that mediate orientation selectivity (Antinucci et al., 2016). Recent studies have implicated alternative splicing of Tenm3 in synapse-specific wiring, and alternative splicing may also be critical for establishing a role in inhibitory synapse formation (Araç and Li, 2019). Whether alternative splicing of Tenm3 plays a postsynaptic role in inhibitory synapse formation remains to be determined.

It is likely that molecular factors within SACs also influence the wiring process. The strongest evidence for this was in the investigation of *FRMD7*, a gene that is associated with congenital nystagmus when mutated. This ailment arises from a deficit in the optokinetic reflex, a behavior strongly associated with DSGCs (Oyster et al., 1972). In the retina, FRMD7 is exclusively expressed by SACs (Yonehara et al., 2016). The *FRMD7-*mutant phenotype is characterized by loss of direction selectivity in horizontal-preferring DSGCs, while preserving direction selectivity in vertical-preferring DSGCs. FRMD7 is an intracellular protein and therefore is not thought to encode for a synaptogenic factor or a cell surface recognition molecule itself but rather is involved in trafficking the critical molecules. Though the exact mechanism is yet to be identified, this directional axis-specific phenotype suggests that a distinct set of SAC proteins could act to differentiate horizontal- from vertical-preferring DSGCs.

### Cbln4 and synapse formation

We pursued one of the strongest differentially expressed genes in the C1q family, *Cbln4*. Cbln4 is one subfamily of proteins (cerebellin 1–4) that are found in subsets of neurons throughout the brain. Cerebellins mediate interactions with distinct isoforms of presynaptic neurexins and postsynaptic receptors that are critical for synaptogenesis (Südhof, 2023). Cbln4 binds to neurexin-1, which is expressed by SACs, and to the receptors DCC, neogenin-1, and possibly GluD1, which are expressed by RGCs (Fig. 3*D*; Tran et al., 2019; Yan et al., 2020; Shekhar et al., 2022). In the cortex, Cbln4 promotes GABAergic synapse formation between SST interneuron dendrites onto pyramidal cell dendrites; shRNA depletion of Cbln4 in SST neurons decreased GABAergic synapse number (Favuzzi et al., 2019; Fossati et al., 2019), while overexpression increased synapse number (Favuzzi et al., 2019). Interestingly, recent studies revealed that targeted KO of Cbln4 from cells in the medial habenula did not impact synapse formation but led to profound deficits in long-term potentiation, which impaired learned behaviors in the mutant mouse (Seigneur et al., 2018; Liakath-Ali et al., 2022). A recent transcriptomic study of cell types in the superior colliculus (SC) found that Cbln4 was strongly expressed in a particular cell type within the SC—an inhibitory direction-selective cell (Liu et al., 2023). These Cbln4+ cells exhibited preferred directions along all axes and represented roughly 50% of the DSGCs in the SC. Whether Cbln4 plays a role in guiding DSGC inputs onto this particular cell or whether it affects the wiring of this SC neuron to its downstream partner remains to be determined.

In our study, we knocked out Cbln4 in RGCs alone with the goal of identifying a possible cell-autonomous role in promoting inhibitory synaptogenesis in ventral-preferring DSGCs. In these mice, Cbln4 is still strongly expressed in the amacrine cell layer (Figs. 3, 4). scRNA-seq data points to VIP+ amacrine cells as a potential source of Cbln4 (Yan et al., 2020). VIP+ amacrine cells are primarily in the INL but also in GCL (Akrouh and Kerschensteiner, 2015; Pérez de Sevilla Müller et al., 2019), and they form synapses with ON–OFF DSGCs and some ON–OFF small-receptive field RGCs (Park et al., 2015; Bleckert et al., 2018). Whether Cbln4 secreted from amacrine cells contributes to synaptic organization among RGCs remains to be determined.

It is unlikely that different cerebellin isoforms could compensate for the loss of Cbln4 from RGCs. Cbln1–3 target different postsynaptic receptors than Cbln4 and exhibit largely nonoverlapping functions during synapse formation (Wei et al., 2012; Zhong et al., 2017; Fossati et al., 2019) and therefore are not likely to developmentally compensate for the loss of Cbln4 in RGCs. Moreover, genetic ablation of Cbln1, Cbln2, and Cbln4 did not lead to synergistic phenotypes consistent with them functioning independently (Seigneur and Südhof, 2018).

Our observation of *Cbln4* mRNA localized to the primary dendrites of *Hb9-GFP* DSGCs suggests that it performs a specific synaptogenic function in the IPL (Fig. 3*B*). However, the RGC types which we found to express Cbln4 also send projections to diverse targets in the SC and lateral geniculate nucleus (Martersteck et al., 2017; Liang et al., 2020; Jiang et al., 2022). As such, it is also possible that *Cbln4* mRNA or translated protein is trafficked to axon terminals of Cbln4+ RGCs to promote central target specificity or to guide feedback connections onto RGC terminals.

In conclusion, our RNA-seq-based screen for genes involved in asymmetric targeting of DSGC dendrites to appropriately oriented SAC processes identified hundreds of candidate molecules involved in cell→cell signaling or synaptogenesis. Although one of these molecules, Cbln4, is known to promote inhibitory synaptic organization in other brain regions, its ablation from RGCs had an insignificant effect on synaptic strength and a small effect on directional tuning among the DSGCs in which it is enriched. This suggests that other synaptic proteins are more important in this asymmetric wiring process, perhaps those that undergo differential splicing like Tenm3 or those that are part of large multi-isoform families like clustered protocadherins.
