# Supplementary figures and images for: Differential Expression Analysis Identifies Candidate Synaptogenic Molecules for Wiring Direction-Selective Circuits in the Retina
Source: J Neurosci. 2024 Mar 21;44(18):e1461232024. doi: 10.1523/JNEUROSCI.1461-23.2024 (PMC11063823; doi:10.1523/JNEUROSCI.1461-23.2024)

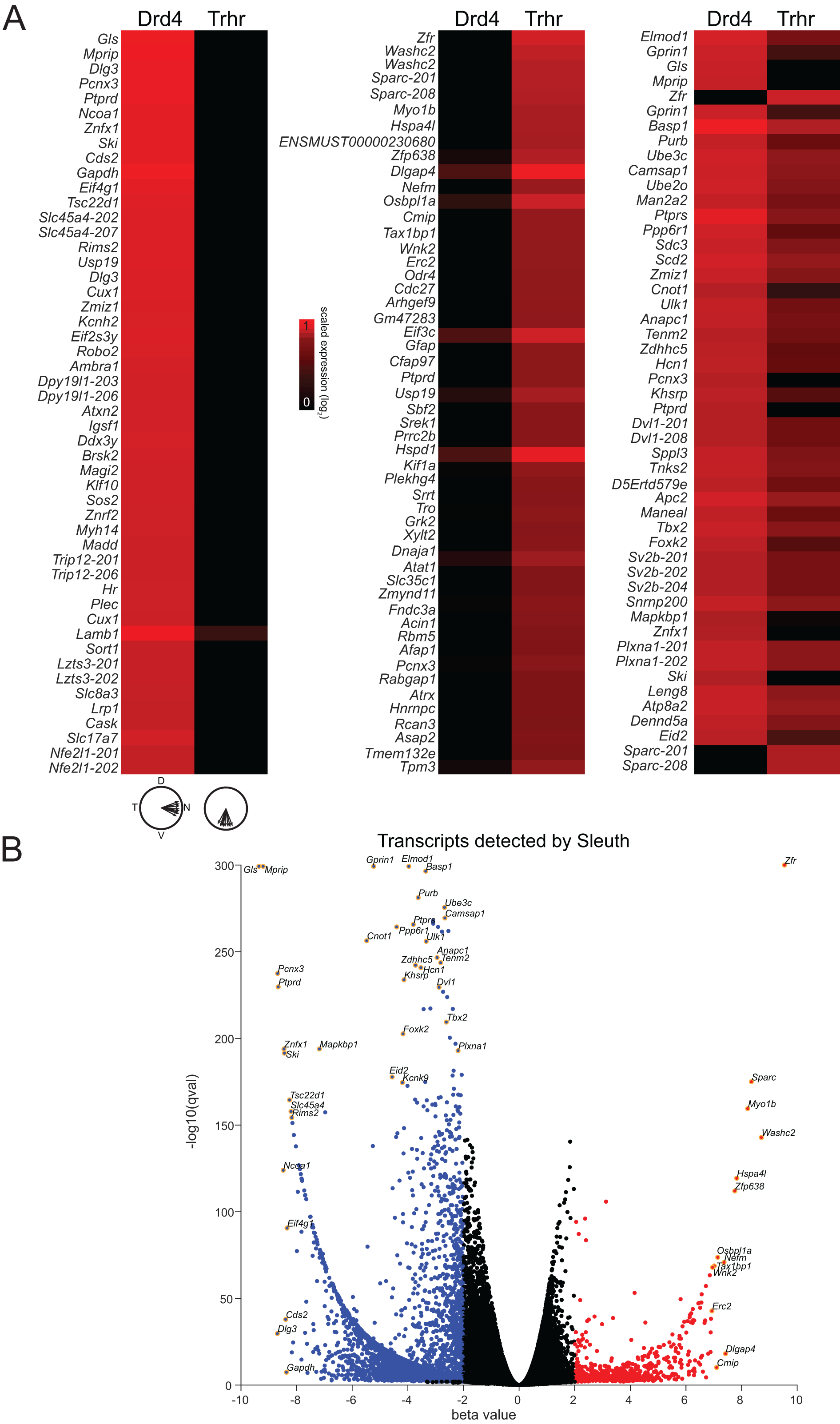

Supplement: Figure 2-1 — Download Figure 2-1, TIF file. [file jneuro-44-e1461232024-s001.tif]

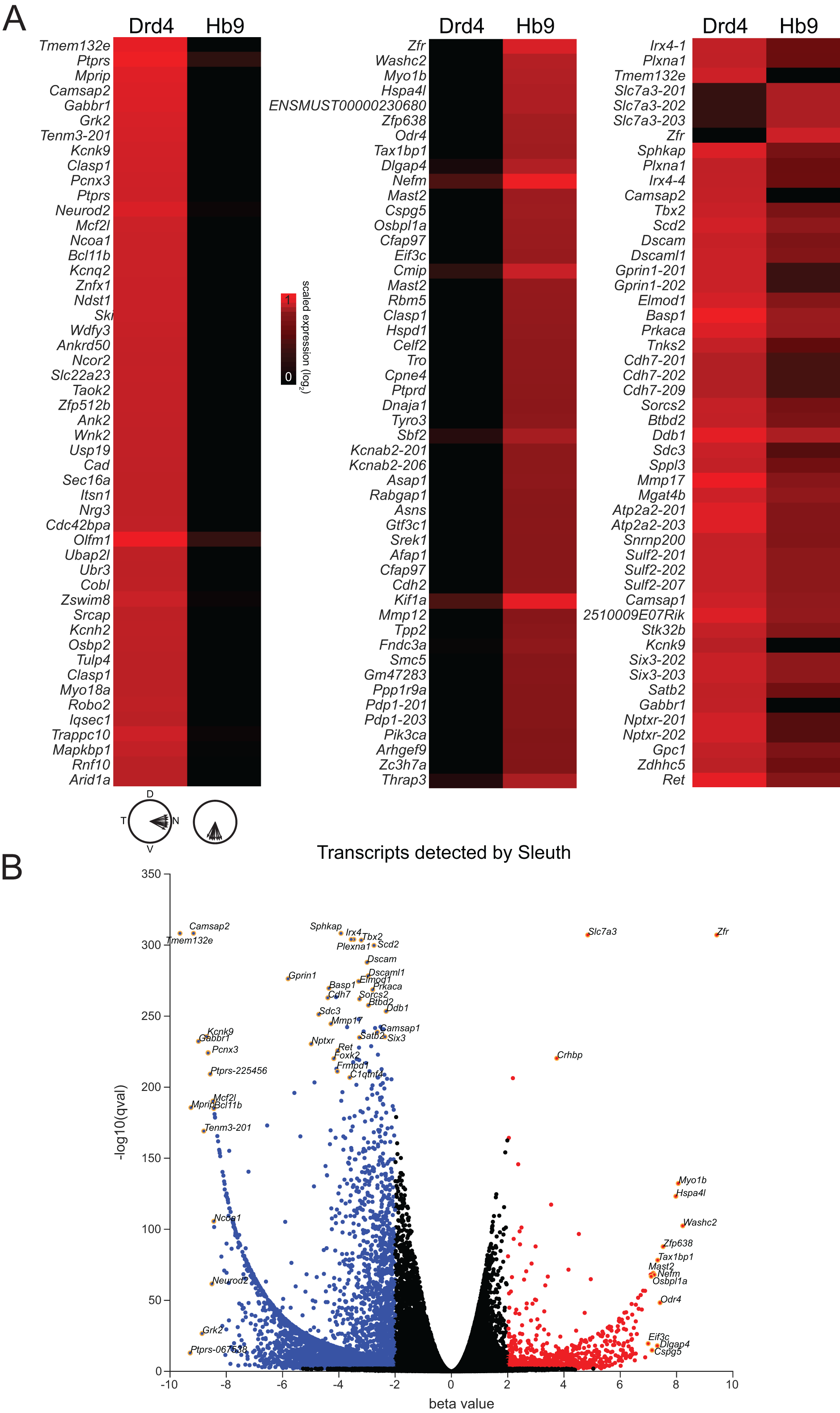

Supplement: Figure 2-2 — Download Figure 2-2, TIF file. [file jneuro-44-e1461232024-s002.tif]

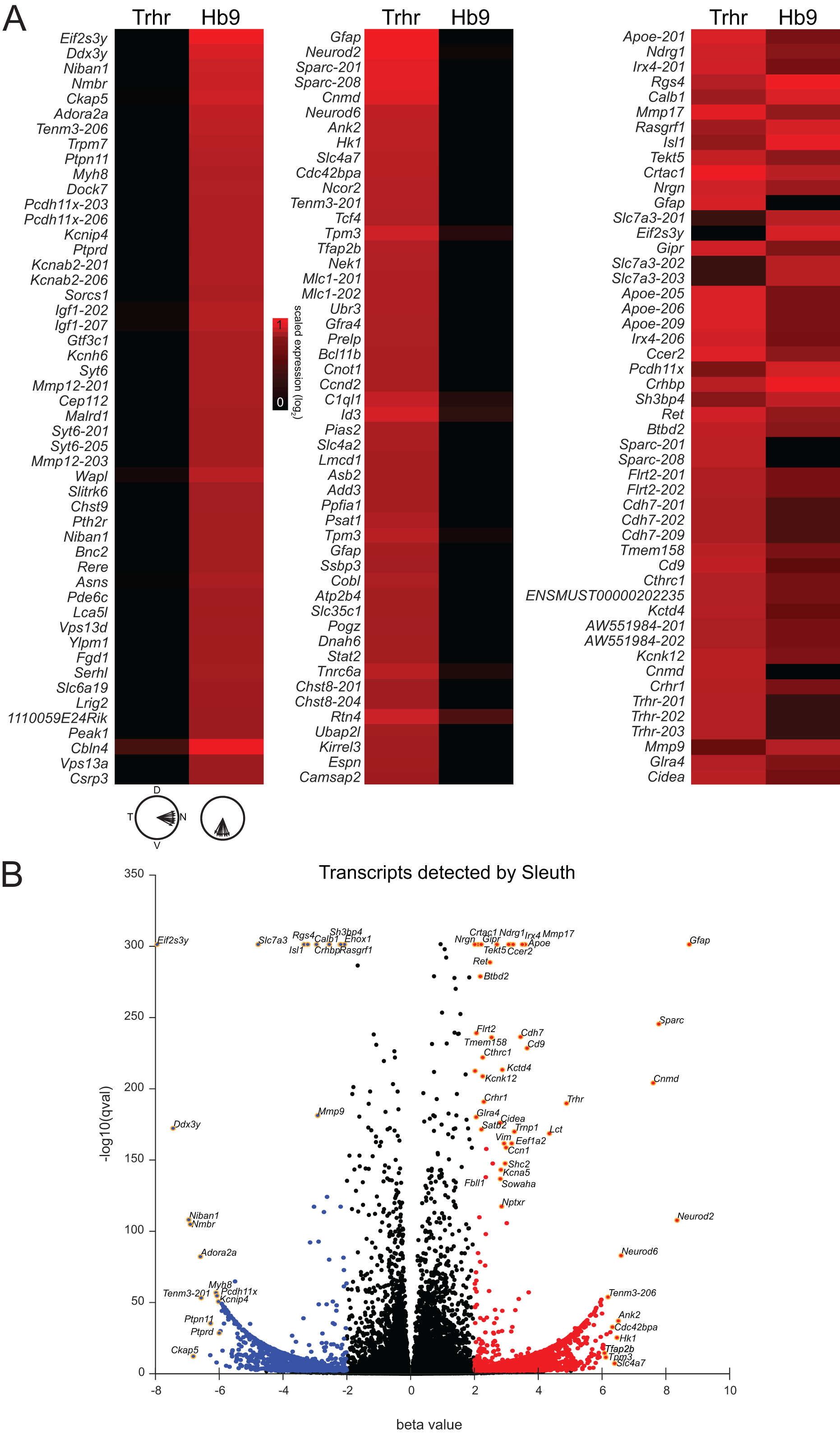

Supplement: Figure 2-3 — Download Figure 2-3, TIF file. [file jneuro-44-e1461232024-s003.tif]

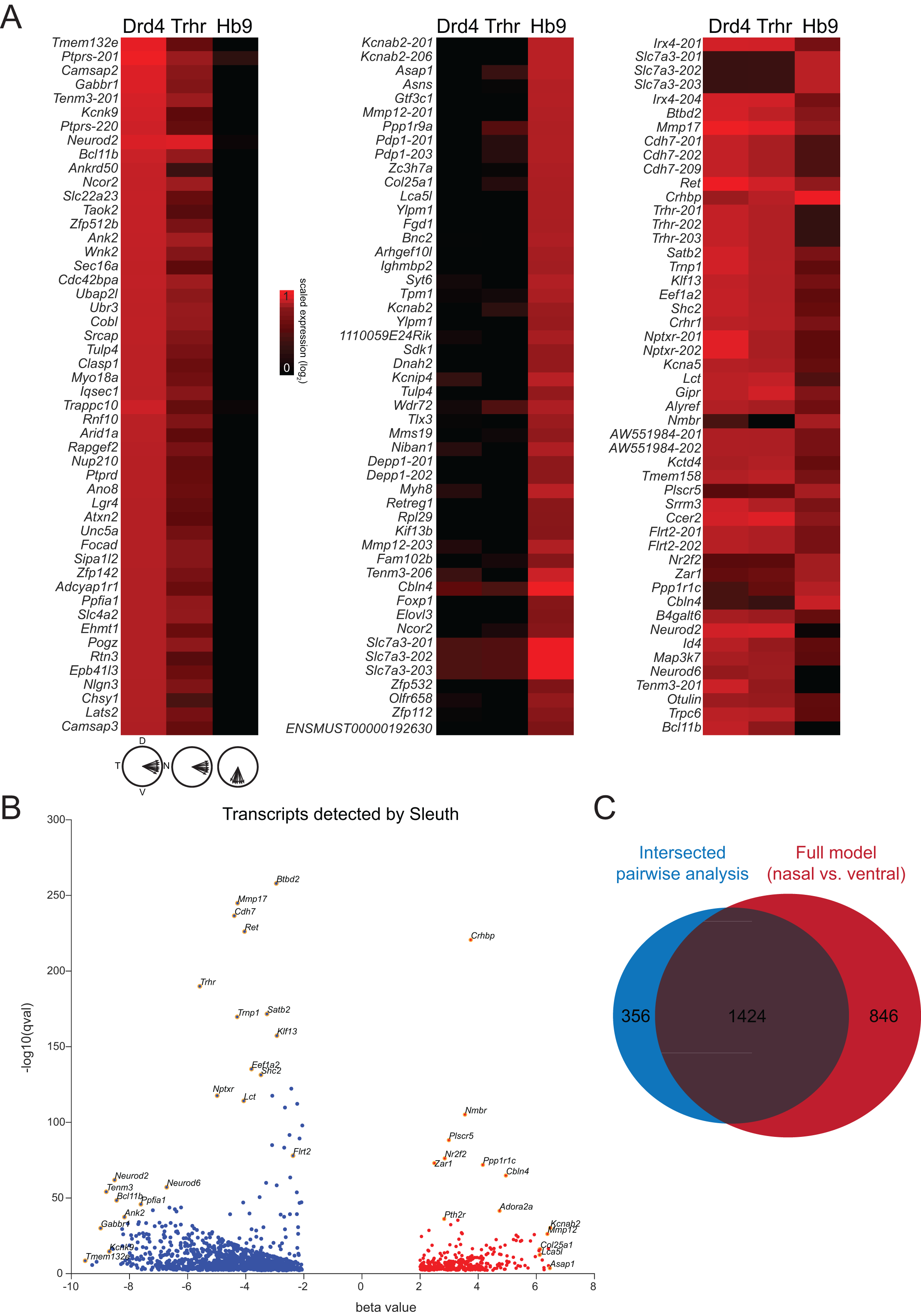

Supplement: Figure 2-4 — Download Figure 2-4, TIF file. [file jneuro-44-e1461232024-s004.tif]

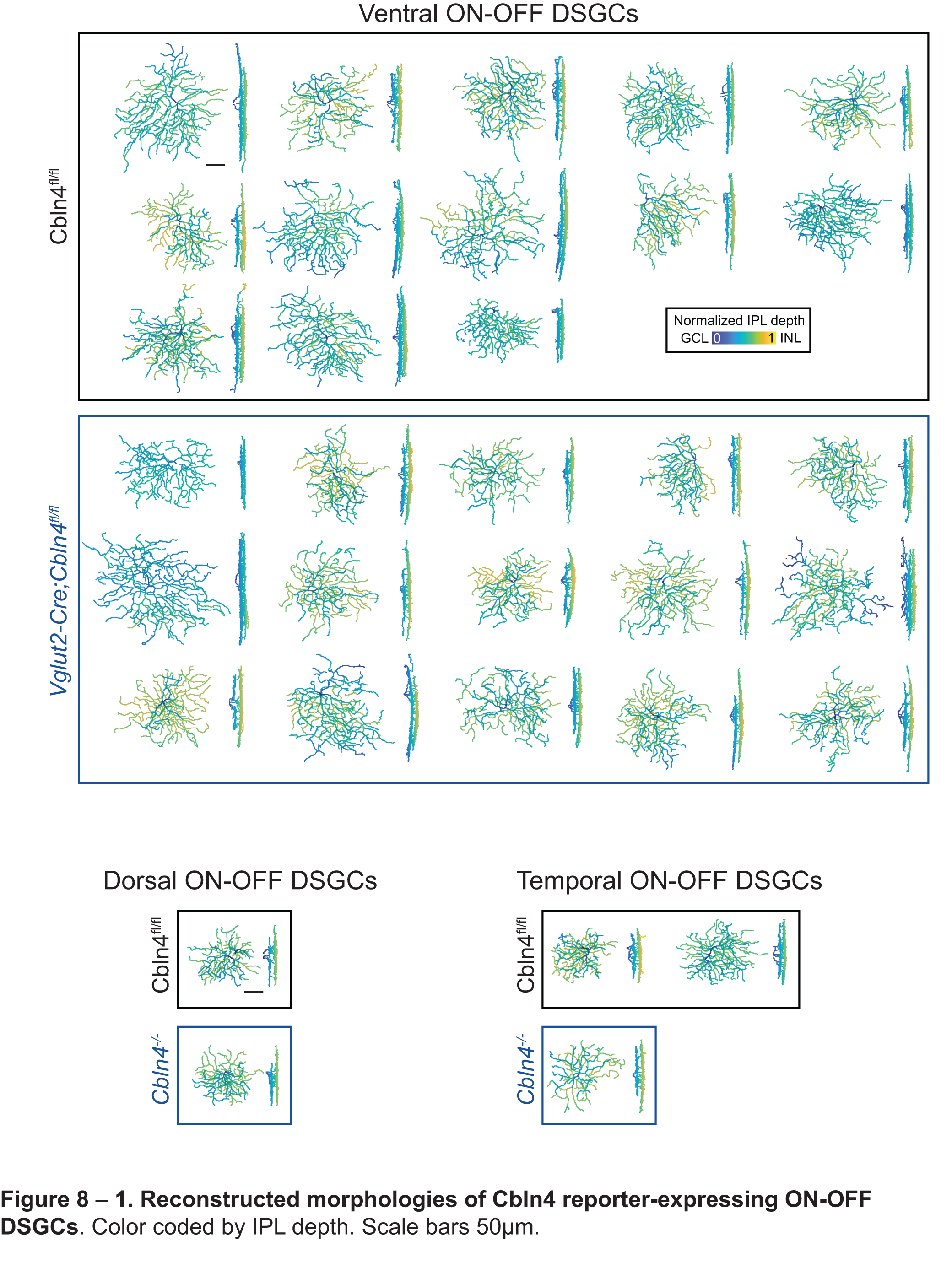

Supplement: Figure 8-1 — Reconstructed morphologies of Cbln4 reporter-expressing ON-OFF DSGCs. Color coded by IPL depth. Scale bars 50μm. Download Figure 8-1, TIF file. [file jneuro-44-e1461232024-s005.tif]

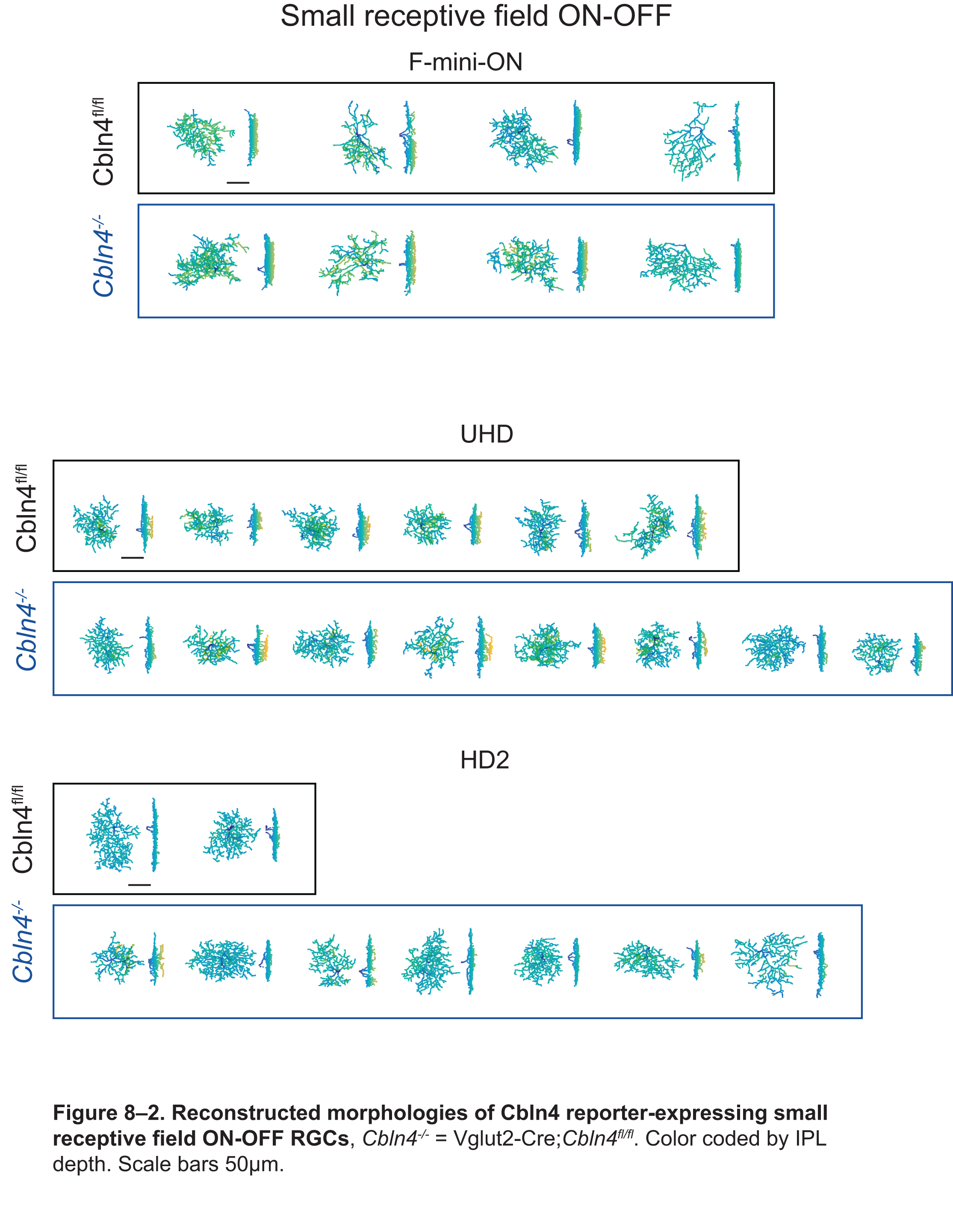

Supplement: Figure 8-2 — Reconstructed morphologies of Cbln4 reporter-expressing small receptive field ON-OFF RGCs. Cbln4-/- = Vglut2-Cre;Cbln4fl/fl. Color coded by IPL depth. Scale bars 50μm. Download Figure 8-2, TIF file. [file jneuro-44-e1461232024-s006.tif]

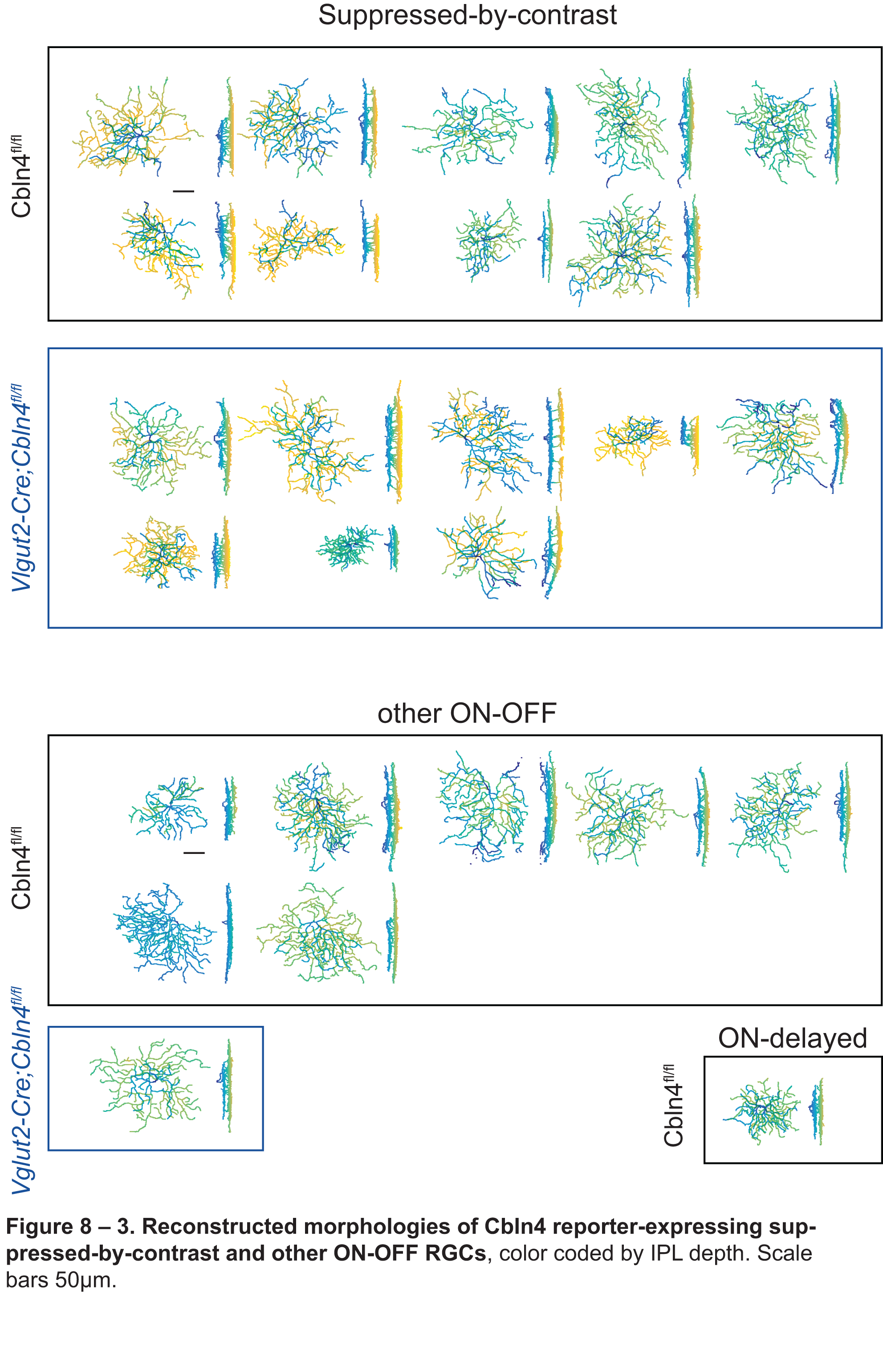

Supplement: Figure 8-3 — Reconstructed morphologies of Cbln4 reporter-expressing suppressed-by-contrast and other ON-OFF RGC. Color coded by IPL depth. Scale bars: 50μm. Download Figure 8-3, TIF file. [file jneuro-44-e1461232024-s007.tif]

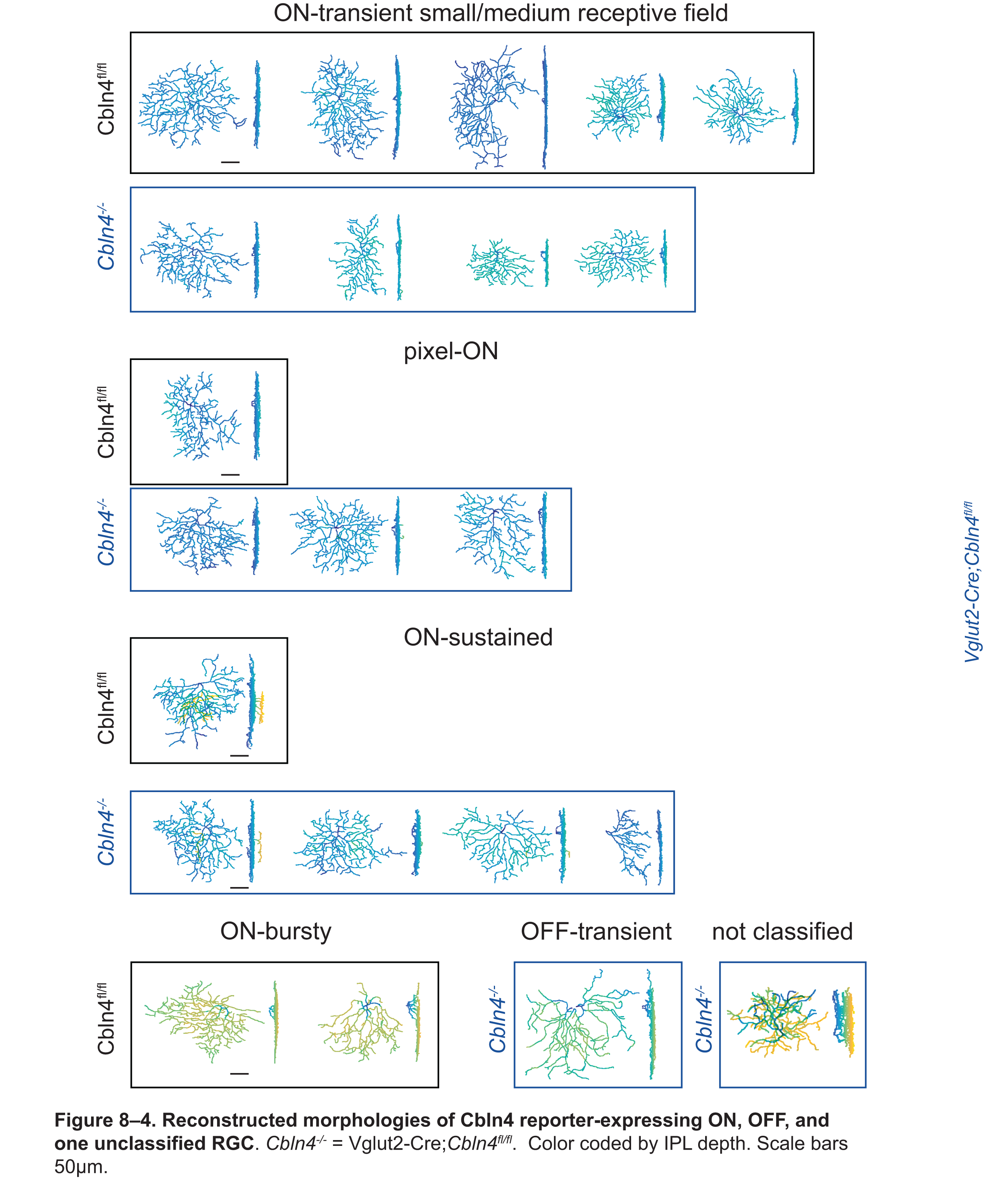

Supplement: Figure 8-4 — Reconstructed morphologies of Cbln4 reporter-expressing ON, OFF, and one unclassified RGC. Cbln4-/- = Vglut2-Cre;Cbln4fl/fl. Color coded by IPL depth. Scale bars: 50μm. Download Figure 8-4, TIF file. [file jneuro-44-e1461232024-s008.tif]
